# Fundamental Understanding of Hydrogen Evolution Reaction on Zinc Anode Surface: A First-Principles Study

**DOI:** 10.1007/s40820-024-01337-0

**Published:** 2024-02-06

**Authors:** Xiaoyu Liu, Yiming Guo, Fanghua Ning, Yuyu Liu, Siqi Shi, Qian Li, Jiujun Zhang, Shigang Lu, Jin Yi

**Affiliations:** 1 Institute for Sustainable Energy & Department of Chemistry, Shanghai University, Shanghai, 200444 People’s Republic of China; 2 School of Materials Science and Engineering, Shanghai University, Shanghai, 200444 People’s Republic of China; 3 College of Materials Science and Engineering, National Engineering Research Center for Magnesium Alloys, Chongqing University, Chongqing, 400044 People’s Republic of China

**Keywords:** Aqueous Zn-ion battery, Zn anode, Hydrogen evolution reaction, Coordination number, First-principles calculation

## Abstract

Hydrogen evolution reaction (HER) has become a key factor affecting the cycling stability of aqueous Zn-ion batteries, while the corresponding fundamental issues involving HER are still unclear. Herein, the reaction mechanisms of HER on various crystalline surfaces have been investigated by first-principle calculations based on density functional theory. It is found that the Volmer step is the rate-limiting step of HER on the Zn (002) and (100) surfaces, while, the reaction rates of HER on the Zn (101), (102) and (103) surfaces are determined by the Tafel step. Moreover, the correlation between HER activity and the generalized coordination number ($\overline{CN }$) of Zn at the surfaces has been revealed. The relatively weaker HER activity on Zn (002) surface can be attributed to the higher $\overline{CN }$ of surface Zn atom. The atomically uneven Zn (002) surface shows significantly higher HER activity than the flat Zn (002) surface as the $\overline{CN }$ of the surface Zn atom is lowered. The $\overline{CN }$ of surface Zn atom is proposed as a key descriptor of HER activity. Tuning the $\overline{CN }$ of surface Zn atom would be a vital strategy to inhibit HER on the Zn anode surface based on the presented theoretical studies. Furthermore, this work provides a theoretical basis for the in-depth understanding of HER on the Zn surface.

**Supplementary Information:**

The online version contains supplementary material available at 10.1007/s40820-024-01337-0.

## Highlights

- The reaction mechanisms of hydrogen evolution reaction (HER) on various crystal surfaces of zinc anode have been systematically investigated by first-principle calculations.
- Both the thermodynamic and kinetic aspects of HER have been studied to reveal the relative HER activity of several crystal surface of zinc anode.
- The generalized coordination number of surface Zn atoms are proposed as a key descriptor of HER activity of Zn anode.

**Supplementary Information:**

The online version contains supplementary material available at 10.1007/s40820-024-01337-0.

## Graphical abstract

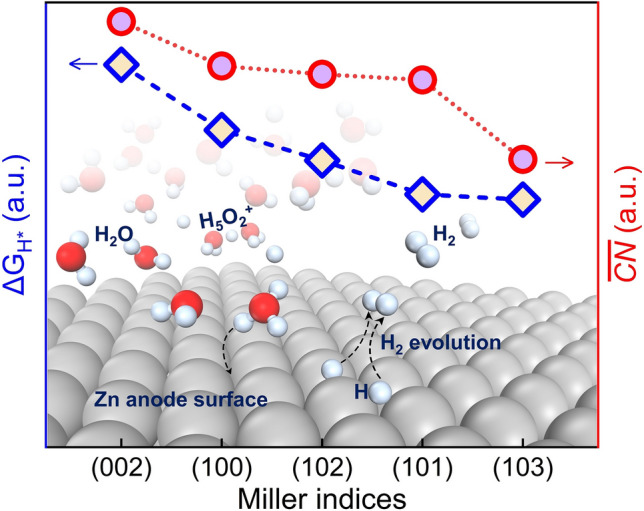

## Introduction

The challenges associated with zinc anode, such as dendrite growth, hydrogen evolution reaction (HER), and passivation, have greatly hindered the practical applications of aqueous Zn-ion batteries (AZIBs) [1–5]. The standard electrode potential of Zn^2+^/Zn (− 0.76 V vs. SHE) in aqueous solution is below the standard electrode potential of HER. The relative potential to initiate Zn^2+^/Zn deposition and HER depends on both the overpotential of HER and Zn deposition. The hydrogen evolution can lead to swelling of AZIBs, which would result in safety issues [6–9]. In addition, the contact between solid phase and liquid phase will be impeded by the H_2_ gas bubbles formed during HER processes, resulting in large polarization potential and even circuit break [10–13]. Meanwhile, the HER will lead to an increase in the pH of the nearby domain [14]. Subsequently, the formation of by-products such as Zn(OH)_2_, Zn_4_(OH)_6_SO_4_⋅*x*H_2_O will take place due to the increase of pH [15], thus passivating the zinc metal and inhibiting the transport of Zn^2+^ [16–18]. The above-mentioned HER-related side reactions will largely affect the electrochemical performance of AZIBs.

Coating and alloying are common modification strategies for Zn anode to inhibit HER [19–24]. Moreover, tuning the exposed crystal surface of Zn anode has been proposed as an effective strategy. The Zn (002) oriented hexagonal texture parallel to the Zn substrate with high stack density would show less corrosion and dissolution, resulting in improved cycling stability [25–27]. Song et al*.* have proposed that Zn (002) texture could be obtained via a one-step annealing process on a commercial Zn foil, which has improved the electrochemical performance of Zn anode [28]. In addition, Hao et al*.* have suggested that the gel electrolyte with sulfonate and imidazole groups could promote Zn deposition with Zn (002) surface exposed, which can suppress HER [29]. The Zn (002) surface has been regarded as the most stable facet against HER [30].

However, the detailed mechanism for the relatively lower HER activity of Zn (002) surface is still unclear. The exposed surface of the primary particles would contain several crystal surfaces, it is difficult to decouple the role of Zn (002) surface via the experimental methods. Fundamentally, the HER activity of a catalyst is determined by the interaction between the intermediates and the catalyst. Sabatier's principle suggests that catalysts with high HER activity must bond with reaction intermediates neither too strongly nor too weakly [31]. The hydrogen adsorption step will be difficult to occur if the hydrogen is too weakly bonded to the surface. The hydrogen liberation step will be limited if the hydrogen is too strongly bonded to the surface [32]. The hydrogen adsorption free energy $\Delta {G}_{{{\text{H}}}^{*}}$ ~ 0 based on first-principles calculations are conventionally used as a criterion of the optimal HER activity [33], which is represented in the “volcano” plots [34]. Since only the $\Delta {G}_{{{\text{H}}}^{*}}$ with 1/4 monolayer coverage is considered in the conventional “volcano” plot, the effect of hydrogen coverage on $\Delta {G}_{{{\text{H}}}^{*}}$ needs to be considered [35]. The *d*-band center is also used to predict the HER activity of transition metals as the $\Delta {G}_{{{\text{H}}}^{*}}$ is correlated with the *d*-band center [36]. However, the dependence between the *d*-band center and HER activity is untenable in some cases. For example, the *d* band center of Ni and Pt are almost the same, while Ni is more inert than Pt in terms of HER. Focusing on the HER activity of the Zn anode, the fully filled *d* bands of Zn make it more difficult to elucidate the relative HER activity of different facets of Zn metal from electronic structures. Therefore, a more specific descriptor to evaluate HER activity at different Zn surfaces and a fundamental understanding of the HER activity are needed, which would yield more specific strategies to inhibit HER at the Zn anode surface.

In this work, the HER activities on several crystal surfaces containing the Zn (002), (100), (101), (102), and (103) surfaces are explored from both the thermodynamic and kinetic aspects via first-principles calculations. The hydrogen adsorption free energy ($\Delta {G}_{{{\text{H}}}^{*}}$) and the activation barrier of Volmer and Tafel steps at the above Zn surfaces have been calculated. The mechanisms of the relatively weaker HER activity on the Zn (002) surface are revealed, which can be attributed to the higher $\overline{CN }$ of surface Zn atom. Once the surface of the Zn (002) slab mode is not flat at an atomic level, the uneven Zn (002) surface would show significantly higher HER activity than the flat Zn (002) surface. The $\overline{CN }$ of the surface Zn atom is proposed as a key descriptor of HER activity. According to the key descriptor of $\overline{CN }$, the most stable adsorption site for the H atom and the HER activity could be predicted. Tuning the $\overline{CN }$ of surface Zn atom would be a vital strategy to inhibit HER on the Zn anode.

## Computational Details

First-principles calculations based on density-functional theory (DFT) in this work were performed in the Vienna ab-initio simulation package (VASP) [37]. The Perdew Burke-Ernzerhof (PBE) [38] pseudopotentials and projector-enhanced wave (PAW) [39] were used. The cutoff energy for the plane-wave basis set was set to 520 eV. The k-points settings are listed in Table S1. Gamma centered grid was applied for Brillouin zone integration. The Zn crystal surfaces were constructed by using the slab model with a symmetric slab containing a vacuum layer (> 15 Å), which can avoid interactions between adjacent slabs. The detailed information of slab models is provided in Table S1 and Fig. S1. The convergence threshold was set as 10^−5^ eV in energy and 0.01 eV/Å in force. The transition state of HER was calculated using the CI-NEB [40] and dimer [41] methods.

The surface energy ($\gamma$) was calculated based on Eq. (1):

1 $$\upgamma =\frac{{E}_{slab}-{E}_{bulk}\times {n}_{slab}}{2\times {A}_{slab}}$$

where *E*_slab_ is the total energy of the slab model, *E*_bulk_ is the average energy per Zn atom in the bulk model of Zn metal, *n* is the number of Zn atoms in the slab model, and *A*_slab_ is the surface area.

The hydrogen adsorption energies ($\Delta {E}_{{{\text{H}}}^{*}}$) is defined as Eq. (2):

2 $$\Delta {E}_{{{\text{H}}}^{*}}=\frac{{E}_{{{\text{nH}}}^{*}}-{E}_{{\text{Zn}}}-\frac{n}{2}{E}_{{{\text{H}}}_{2}}}{n}$$

where ${E}_{{{\text{nH}}}^{*}}$ is the total energy of the slab with *n* H atoms adsorbed, ${E}_{Zn}$ is the energy of the Zn slab model, and ${E}_{{{\text{H}}}_{2}}$ is the energy of the H_2_ molecule in the gas phase state.

The hydrogen adsorption free energies ($\Delta {G}_{{{\text{H}}}^{*}}$) were calculated based on Eq. (3):

3 $$\begin{aligned}\Delta {G}_{{{\text{H}}}^{*}}&=\Delta {E}_{{{\text{H}}}^{*}}+\Delta ZPE-T\Delta S\\&=\Delta {E}_{{{\text{H}}}^{*}}+{ZPE}_{{{\text{H}}}^{*}}-{\frac{1}{2}{\text{ZPE}}}_{{{\text{H}}}_{2}}-T{S}_{{{\text{H}}}^{*}}+{\frac{1}{2}TS}_{{{\text{H}}}_{2}}\end{aligned}$$

where the $\Delta {E}_{{{\text{H}}}^{*}}$ is defined in Eq. (2). The temperature is set to 300 K. The ${ZPE}_{{{\text{H}}}^{*}}$, ${S}_{{{\text{H}}}^{*}}$, ${ZPE}_{{{\text{H}}}_{2}}$, and ${S}_{{{\text{H}}}_{2}}$ are the zero-point vibration energy and entropy of H that adsorbed on Zn surface and gas phase H_2_ under standard atmospheric pressure, respectively.

The generalized coordination number ($\overline{CN }$) of the surface Zn atom is defined as Eq. (4):

4 $$\overline{CN }\left(i\right)=\sum_{j=1}^{{n}_{i}}\frac{cn\left(j\right)}{{cn}_{max}}$$

where $cn\left(j\right)$ is the coordination number of zinc atoms adjacent to zinc and ${cn}_{max}$ is the maximum atomic coordination number of Zn atom in Zn metal [42].

## Results and Discussion

### Hydrogen Adsorption at Zn Surfaces

**Fig. 1:**
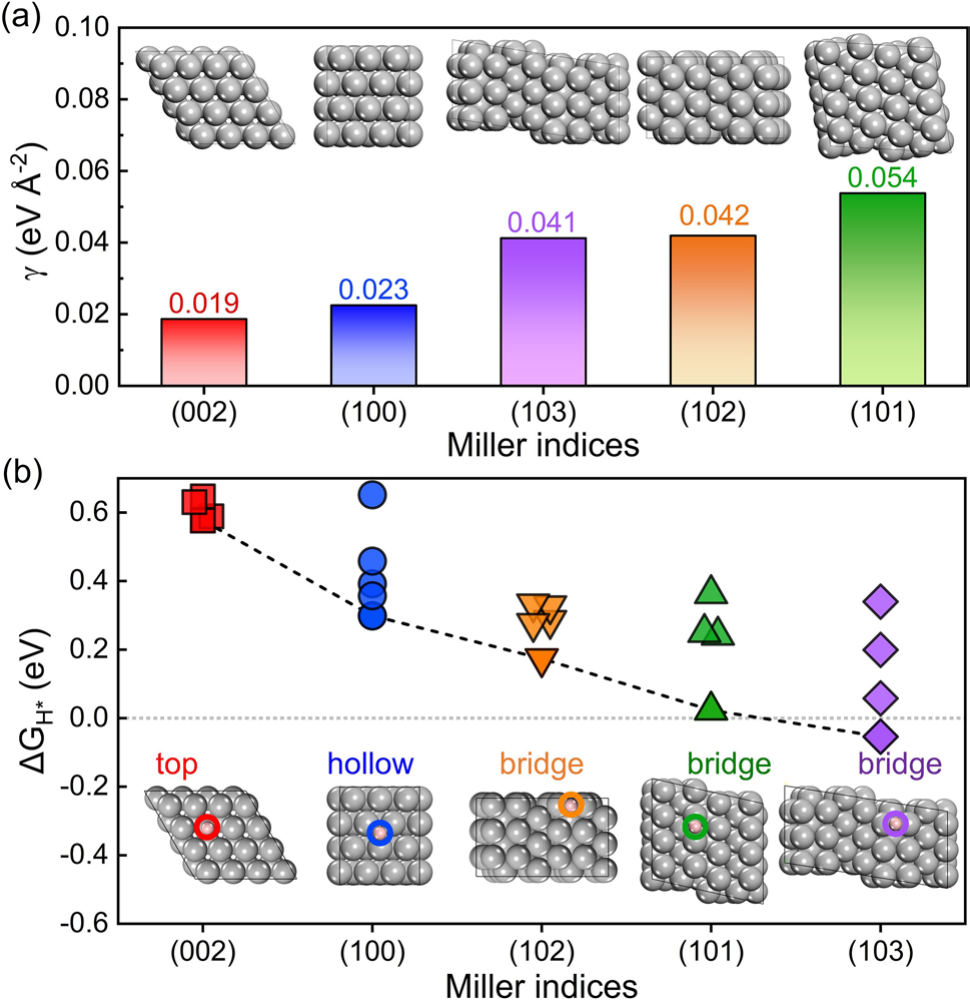
**a** The surface energies of the crystal surfaces of Zn metal with different Miller indices. **b** The $\Delta {G}_{{{\text{H}}}^{*}}$ for H atom adsorbed at different sites of the crystal surfaces of zinc metal

The HER activity on different surfaces of the Zn anode is determined by the intrinsic properties of Zn surface. It is necessary to investigate the properties of different Zn surfaces. Zn metal presents a typical hexagonal close-packed (hcp) structure. The optimized structures of Zn (002), (100), (101), (102), and (103) surfaces are shown in the inserts of Fig. 1a. The Zn (002) surface shows the close-packed arrangement within the surface. Other surfaces yielded structural rearrangement to some extent during the structural optimization processes. Especially, the uneven Zn (100) surface becomes flat surface at an atomic level after optimization. Consequently, the optimized Zn (002) and (100) surfaces are flat at an atomic level, while the optimized Zn (101), (102), and (103) surfaces are uneven at an atomic level.

The surface energies of the above crystal surfaces are calculated, as shown in Fig. 1a. In general, the surface energies are agreement with the database provided in the materials project [43], implying that the structural optimization of the above crystal surfaces of Zn metal is rational. It can be seen that the surface energy of the (002) surface is the lowest, indicating that (002) is more stable than other surfaces. The surface energy of the (100) surface is much lower when compared to that of the (101), (102), and (103) surfaces. Atomically flat surfaces are more stable than uneven surfaces. The crystal surfaces with higher surface energy are expected to present higher reaction activity for oxidation or corrosion [44]. In contrast, the crystal surfaces with lower surface energy are expected to show better stability against interface reactions.

The hydrogen-adsorption free energy ($\Delta {G}_{{{\text{H}}}^{*}}$) is used to predict the HER activity of catalysts from a thermodynamic aspect. According to Sabatier's principle [31], a good HER activity of catalysts need to bond with the H atom neither too strongly nor too weakly. If the H atom bonded to the surface too weakly, the H adsorption step (Volmer) will be difficult to take place. In contrast, the desorption (Heyrovsky/Tafel) step will hard to be proceed when H bonded to the surface too strongly [32]. $\Delta {G}_{{{\text{H}}}^{*}}$ ~ 0 is regarded as the optimal value for good HER activity from the thermodynamic point of view [45]. Thus, $\Delta {G}_{{{\text{H}}}^{*}}$ at different site of Zn (002), (100), (101), (102), and (103) surfaces were calculated to insight into the thermodynamic aspect of HER activity of Zn anode, as shown in Fig. 1b. In addition, Van der Waals interactions with Grimme's D3 scheme have been tested for hydrogen-adsorption models (Fig. S2). The $\Delta {G}_{{{\text{H}}}^{*}}$ values are slightly changed, while the relative values of $\Delta {G}_{{{\text{H}}}^{*}}$ for several crystal surfaces are almost unchanged after considering the Van der Waals interactions. The most stable adsorption site for each crystal surface is identified, which is presented in the insert of Fig. 1b. The adsorption energies of H atoms at bridge sites on the (101), (102), and (103) surfaces are significantly lower than those at other sites, suggesting that the bridge sites are the most stable adsorption site for (101), (102), and (103) surfaces. The detailed $\Delta {G}_{{{\text{H}}}^{*}}$ values and the corresponding structures of H adsorbed at (101), (102), and (103) surfaces are shown in Tables S2–S4 in the supporting information, respectively. And H prefers to be absorbed on the top and hollow sites of (002) and (100) surfaces, respectively. All the adsorption sites and the correspongding $\Delta {G}_{{{\text{H}}}^{*}}$ values at (002) and (100) surfaces that were considered here are shown in Tables S5, S6 in the supporting information, respectively. However, the bridge site adsorption becomes more stable as the H coverage increases, which will be discussed in the following part. The lowest value of $\Delta {G}_{{{\text{H}}}^{*}}$ for the Zn (002) surface is the highest one among several crystal surfaces. The Zn (002) and (100) surfaces show higher $\Delta {G}_{{{\text{H}}}^{*}}$ for the most stable adsorption site than (101), (102), and (103) surfaces. Keep in mind that the above values of $\Delta {G}_{{{\text{H}}}^{*}}$ are calculated at a low hydrogen coverage, and the hydrogen coverages are not the same for different crystal surfaces of Zn metal. Besides, H_2_O adsorption energies on several crystal surfaces have been calculated (Fig. S3). The adsorption energy differences are within 0.1 eV. Generally, the trends of H_2_O adsorption energies are similar to the H adsorption energies. The (002) surface shows the highest H_2_O adsorption energy, indicating that H_2_O molecules are less likely to adsorbed at (002) surface.

**Fig. 2:**
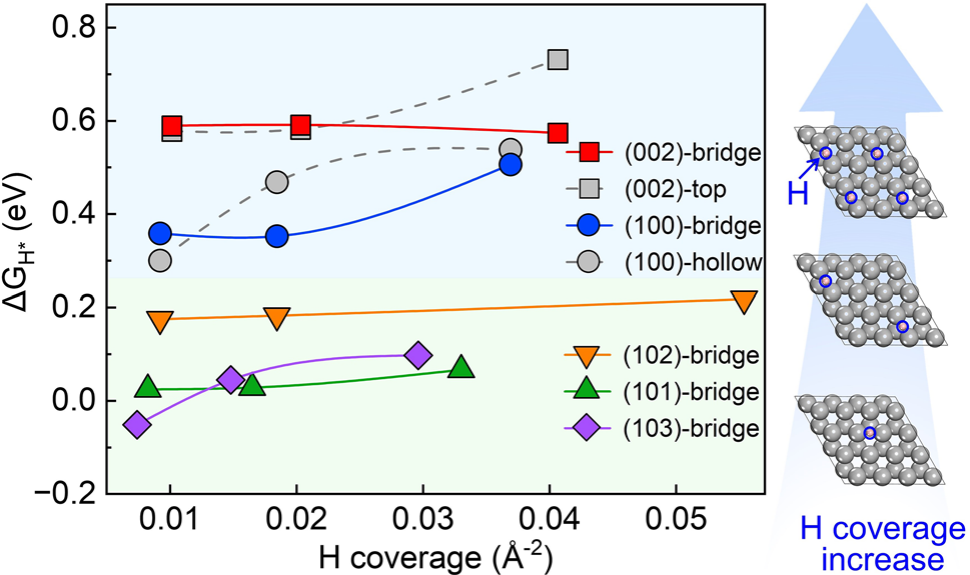
Hydrogen adsorption energy ($\Delta {G}_{{{\text{H}}}^{*}}$) at several crystal surfaces of the zinc anode as a function of different hydrogen coverage. The H coverage indicates the number of hydrogens absorbed per unit area

Considering that the hydrogen coverage presents a significant impact on the hydrogen-adsorption energy on other catalyst surfaces like Pt (111) surface [46, 47], the $\Delta {G}_{{{\text{H}}}^{*}}$ and $\Delta {E}_{{{\text{H}}}^{*}}$ as a function of hydrogen coverage was further investigated, as shown in Figs. 2 and S4, respectively. The details for thermal corrections to Gibbs free energies are listed in Tables S7, S8. The most stable adsorption sites (i.e. the bridge sites) of (101), (102), and (103) surfaces are included. Both the bridge and top sites of (002) crystal face have been conducted because of the small difference of ∆*G*_H*_ between the two sites at a low hydrogen coverage. Similarly, both the bridge and hollow sites of (100) crystal face have been included due to the small difference of ∆*G*_H*_. The H adsorption energies increase as coverage increases. The relative values of adsorption energy between Zn (002), (100), (101), and (102) surfaces at a certain hydrogen coverage are similar. As for the Zn (103) surface, the adsorption energy increases more significantly than the other surfaces. The Zn (103) surface shows the lowest ∆*E*_H_ at low hydrogen coverage, however, the ∆*E*_H_ of the (103) surface becomes higher than that of the (101) surface as the hydrogen coverage increases. The Zn (002) surface presents the highest adsorption energy, and Zn (100) surface takes the second place. The Zn (101), (102), and (103) surfaces show relatively lower adsorption energies than Zn (002) and (100) surfaces. We found that the most remarkable difference between the former surfaces (the Zn (101), (102), and (103) surfaces) and the later surfaces (the Zn (002) and (100) surface) is whether the surface is flat at an atomic level. The flat surfaces tended towards higher adsorption energies, which will be discussed quantitatively later. The Zn (002) surface is expected to be the most stable surface against HER from the thermodynamic point of view. Thus, the relative hydrogen adsorption energies for different crystal surfaces of Zn anode at various hydrogen coverages are clarified, which provides a systematic thermodynamic understanding of the HER activity on each crystal surface of Zn anode.

### HER Kinetic Steps at Zn Surfaces

The HER activity cannot be determined just by the thermodynamic perspective. The challenge to the $\Delta {G}_{{{\text{H}}}^{*}}$ ~ 0 explanation of HER has been proposed by Peterson and co-workers [45]. The dynamic aspects of HER were further studied. HER is a classical two-electron-transfer reaction that can be carried out by either the Volmer–Heyrovsky or the Volmer–Tafel mechanism [48].

In an acidic solution, proton and electron are transferred to the surface via the Volmer step is defined as Eq. (5) [49]:

5 $${\text{H}}^{+} + {\text{ e}}^{-} +^{*} \to {\text{H}}^{*} \, \left( {\text{Volmer}} \right)$$

**Fig. 3:**
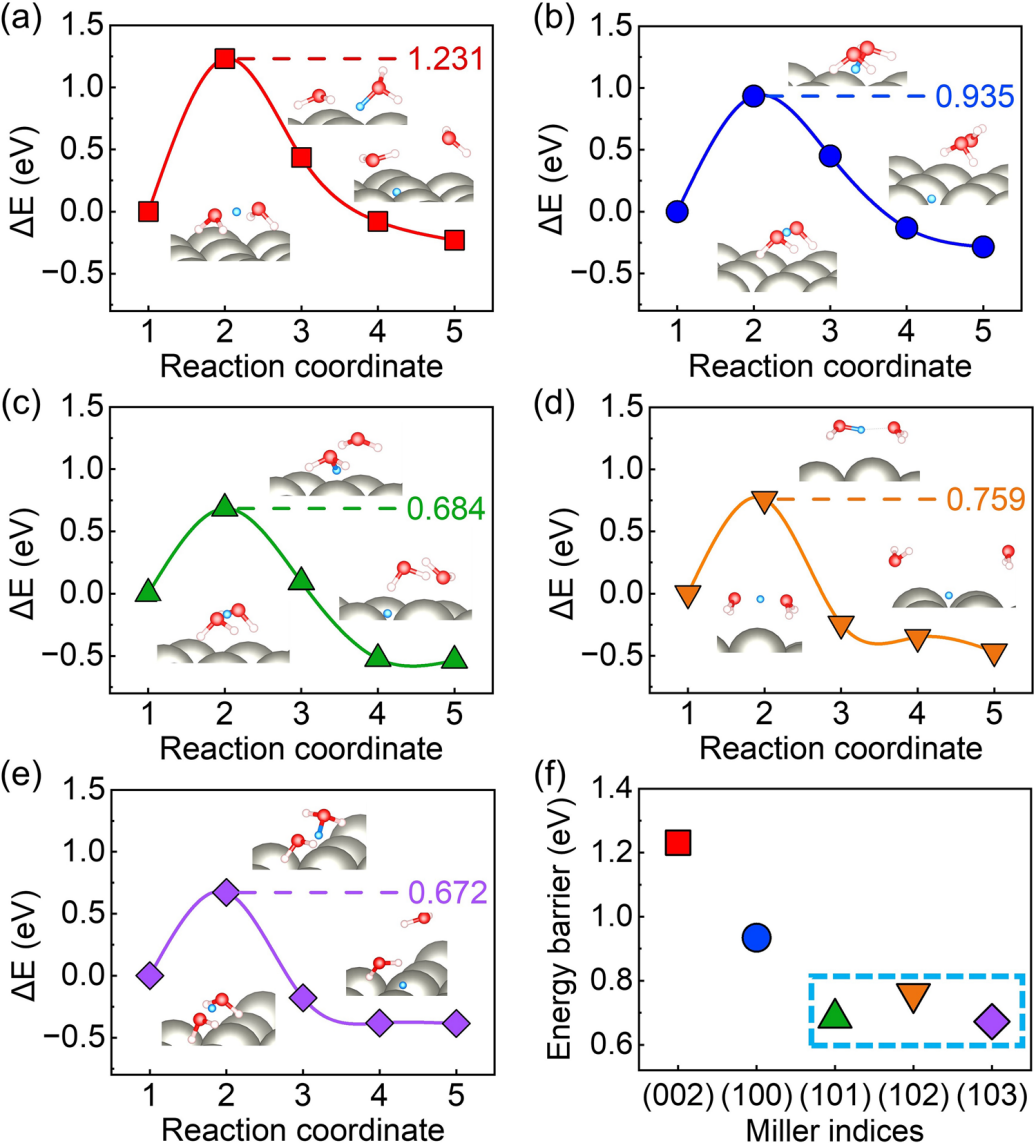
The energy barriers and reaction pathways of Volmer step on several crystal surface of Zn anode. **a** Zn (002) surface, **b** Zn (100) surface, **c** Zn (101) surface, **d** Zn (102) surface, **e** Zn (103) surface, **f** the comparative Volmer reaction barriers. The insets are the structures of corresponding initial, transition, and final states. The white, red, and grey spheres are H, O, and Zn atoms, respectively. The blue spheres are the protons that are willing to adsorb at the surfaces

In order to simulate the Volmer reaction, the Zundel-type hydrated proton (H_5_O_2_^+^) [50] is used as the proton donor. The H_5_O_2_^+^ lay parallelly at the Zn (002) and (100) surfaces with a distance of about 3 Å. And the H_5_O_2_^+^ is oblique to the (101), (102) and (103) surfaces. Figure 3 illustrates the reaction pathway of the Volmer step. The protons are adsorbed at the site with the lowest adsorption energy on the Zn surface after being released from H_5_O_2_^+^. Meantime, the two H_2_O molecules move to the solution. This is an exothermic process (Fig. 3). An activation barrier up to 1.231 eV is delivered between the initial and transition states for the Volmer step on the (002) surface. The (100) surface also shows a relatively high activation barrier of 0.935 eV. The activation energy barriers of the (101), (102), and (103) surfaces are closer to each other, which are significantly lower than those of the (002) and (100) surfaces. The electrostatic potentials of several crystal surface model are visualized in Fig. S5. The more positive values of electrostatic potential for (101), (102), and (103) surfaces indicate that these surfaces are favorable for electron transfer. Thus, the Volmer step would be easier for (101), (102), and (103) surfaces. The Volmer step at Zn (002) surface would be the most difficult to proceed. Furthermore, the variation trends of the energy barrier values of the Volmer step and the hydrogen adsorption energy $\Delta {G}_{{{\text{H}}}^{*}}$ on different crystal surfaces in Fig. 2 are similar. It further proves the correlation between the adsorption energy $\Delta {G}_{{{\text{H}}}^{*}}$ and the kinetic of the Volmer reaction.

**Fig. 4:**
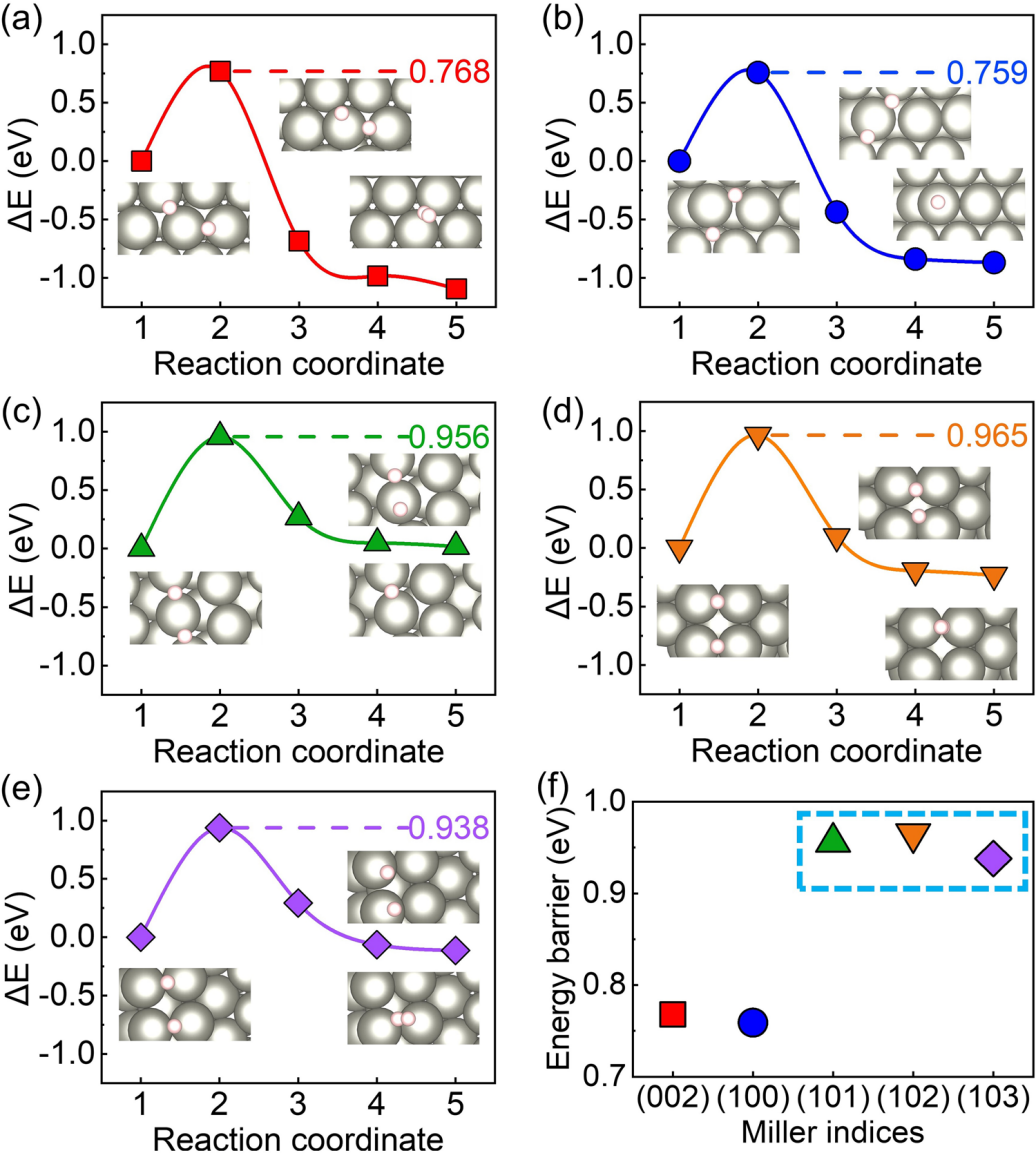
The energy barriers and reaction pathways of Tafel step on several crystal surface of Zn anode. **a** Zn (002) surface, **b** Zn (100) surface,** c** Zn (101) surface, **d** Zn (102) surface, **e** Zn (103) surface,** f** the comparative Tafel reaction barriers. The insets are the structures of corresponding initial, transition, and final states. The white and grey spheres are H and Zn atoms, respectively

The surface-bound hydrogen formed in the Volmer step would undergo a H_2_ liberation step. The Tafel steps (2H* → H_2_) show lower energy barriers than Heyrovsky steps (H* + H^+^  + e^−^  → H_2_) (Fig. S6), thus we focused on Tafel reaction steps. The desorption of two nearby H atoms forms a H_2_ molecule with a bond length of ~ 0.75 Å and a distance of > 3.5 Å from the surface. The similar adsorption energies of H_2_ at different surface range from − 0.02 to 0.01 eV, which is approximately 0 eV, indicating weak physical adsorption between the H_2_ molecule and the surfaces. Figure 4 shows the reaction pathways and the activation barriers of the Tafel reactions at different Zn surfaces. The Zn (101), (102), and (103) surfaces that are uneven at an atomic level show similar energy barriers, which is higher than the Zn (002) and (100) surfaces, indicating that the reaction rate of Tafel step would be slower for Zn (101), (102), and (103) surfaces.

Combining the activation barriers of the Volmer and Tafel steps, it is found that the Volmer step is the rate-limiting step of HER for the Zn (002) and (100) surfaces, while the Tafel step turned to the rate-limiting step for Zn (101), (102), and (103) surfaces. The overall reaction barrier of HER at the Zn (002) surface is significantly higher than that of HER at the Zn (100), (101), (102), and (103) surfaces. The Zn (002) surface is expected to be the most stable surface against HER from both the thermodynamic and dynamic point of view, which is consistent with previous experimental results [51–53].

### Relationship Between $\overline{{\varvec{C}}{\varvec{N}} }$ and HER Activity

As is mentioned above, the most remarkable difference between the former surfaces (the Zn (101), (102), and (103) surfaces) and the later surfaces (the Zn (002) and (100) surface) is whether the surface is flat at an atomic level. Considering the similar hydrogen adsorption energies and activation barriers of HER at Zn (101), (102), and (103) surface, the mechanisms of the difference between the atomically flat surfaces and uneven surfaces are further analyzed. The quantitative descriptor for the qualitative features of the atomically flat and uneven surfaces should be identified.

The Zn atoms at the surface show unsaturated coordination, and thus exhibit the activity to adsorb atoms, ions, and molecules. The upper Zn atoms at the atomically uneven surfaces would present a lower nearest neighbor coordination number (*CN*) than the Zn atoms at the atomically flat surfaces. Indeed, as mentioned above in Fig. 1b and Tables S2–S6 in the supporting information, the upper Zn atoms of uneven (101), (102), and (103) surfaces show lower hydrogen adsorption energies, which is the most stable adsorption site and the active site for HER. The *CN* of the surface Zn atom is expected as a quantitative descriptor of the difference between the atomically flat and uneven surface. However, *CN* for the Zn atom at the (100), (101), and (102) surfaces are the same, which cannot interpret the relative H adsorption energies. Extended from conventional *CN*, the generalized coordination number ($\overline{CN }$) proposed by Calle-Vallejo [42] and co-workers could identify the atomic arrangement of the surface atoms more precisely by considering the coordination number of their nearest neighbors. The $\overline{CN }$ has been proposed to make a fast prediction of adsorption properties for platinum nanoparticles. Thus, the $\overline{CN }$ was considered as the quantitative descriptor of the difference between the atomically flat and uneven surface.

**Fig. 5:**
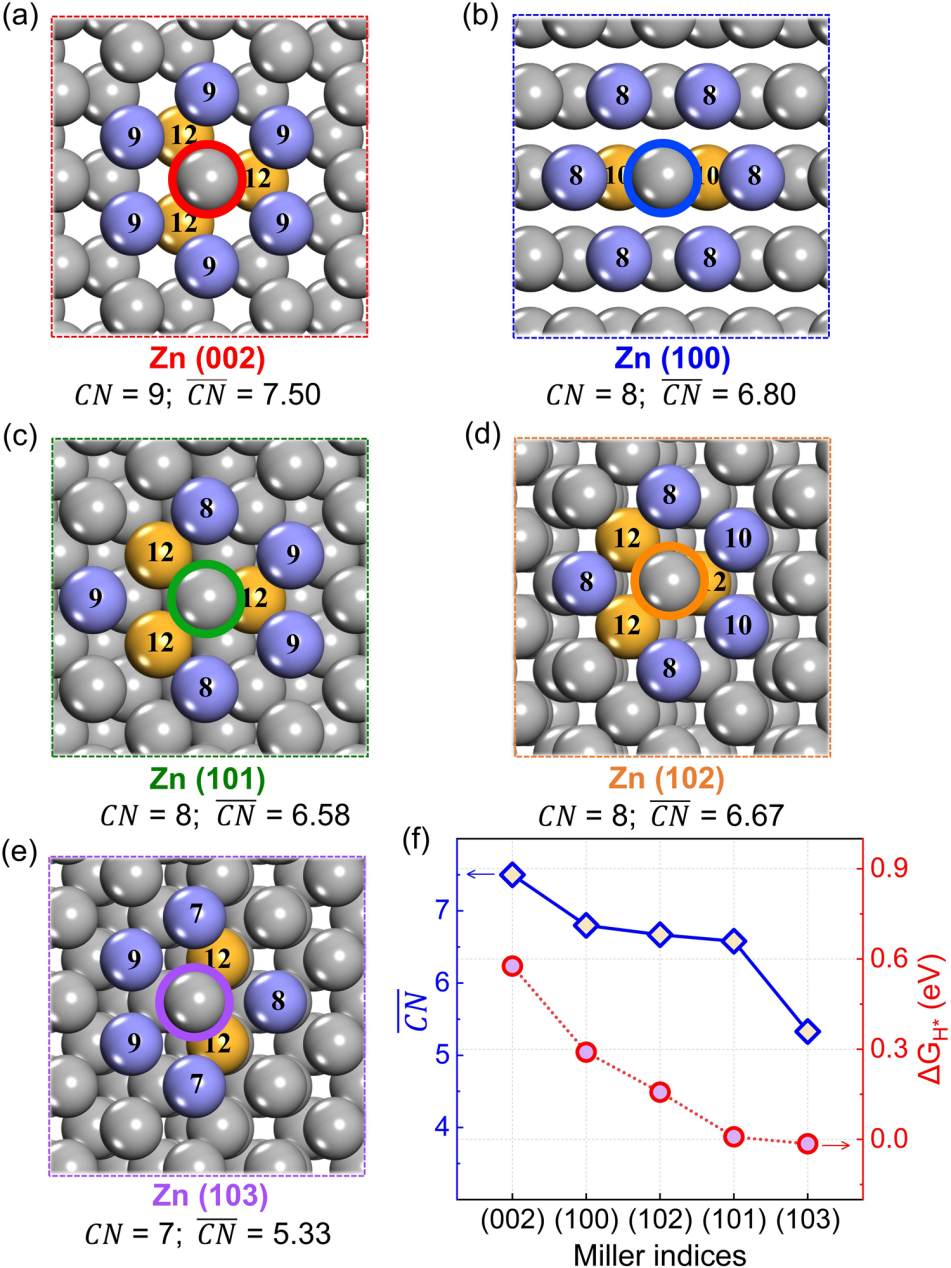
The schematic diagram of the $\overline{CN }$ of Zn atom at several crystal surface of Zn anode. **a** Zn (002) surface, **b** Zn (100) surface, **c** Zn (101) surface, **d** Zn (102) surface, **e** Zn (103) surface, **f** the correlation between the hydrogen adsorption energy and the $\overline{CN }$

The $\overline{CN }$ of Zn atom at the site with the lowest hydrogen adsorption energy was calculated, as shown in Fig. 5. The atomically flat (002) surface shows the largest $\overline{CN }$, and the atomically flat (100) surface takes the second place. The (101), (102), and (103) show relatively lower $\overline{CN }$. As shown in Fig. 5f, there is a correlation between $\overline{CN }$ and the lowest adsorption energy of each surface. The adsorption energy increases with the increase of $\overline{CN }$, which also suggests that the concept of $\overline{CN }$ is also applicable among transition metal Zn. It is also worth noting that H atoms tend to be adsorbed near the Zn with low $\overline{CN }$. A higher $\overline{CN }$ of Zn atoms on crystal surface of Zn anode would deliver higher $\Delta {G}_{{{\text{H}}}^{*}}$, which lead to poor HER activity. Zinc atom in the bulk of zinc metal crystal follows a face centered cubic arrangement, whose $\overline{CN }$ is 12. The $\overline{CN }$ of Zn atom at crystal surface with unsaturated coordination is lower than 12, which tends to form new bonds to compensate for the absence of coordination atoms. Thus, lower $\overline{CN }$ would lead to relatively stronger interaction between H and Zn atoms, indicating lower hydrogen adsorption energy and higher HER activity. The relationship between generalized coordination number ($\overline{CN }$) and HER activity of Zn anode have been revealed. The different $\overline{CN }$ of surface Zn atoms would lead to differences in charge distribution. The correlation between the Bader charge and $\overline{CN }$ have been provided (Fig. S7 and Table S9). The Bader charge decreases as the $\overline{CN }$ increases. Besides, the differential charge density induced by H adsorption for several crystal surfaces are shown in Fig. S8. The differences of Bader charge and differential charge density for several crystal surfaces are very small, which might be caused by the completely filled *d*-bands of Zn atom. Thus, it is hard to explain the HER activity by the charge density.

**Fig. 6:**
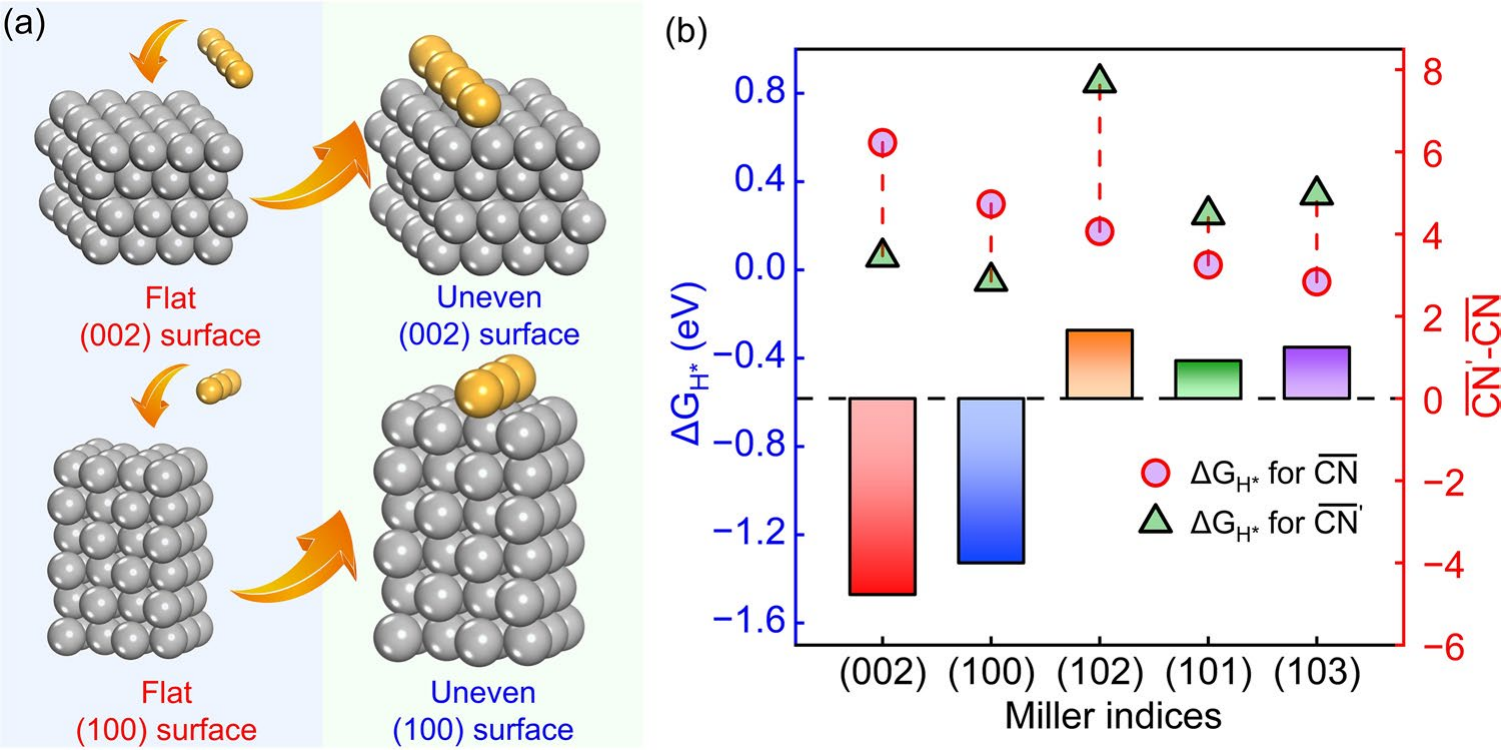
** a** Schematic diagram of uneven (002) and (100) surface. **b** Hydrogen adsorption energy of H adsorbed on surface Zn atom with different $\overline{CN }$

In order to further verify the correlation between $\overline{CN }$ and adsorption energy, the $\overline{CN }$ and adsorption energies for different adsorption sites of the same crystal surfaces have been further studied. As for uneven surface, including (101), (102), and (103) surfaces, the $\overline{CN }$ of Zn atoms vary within the same crystal surface. The $\Delta {G}_{{{\text{H}}}^{*}}$ for adsorption sites with higher $\overline{CN }$ are higher (Figs. 6b and S9), which is consist with the relationship between $\overline{CN }$ and $\Delta {G}_{{{\text{H}}}^{*}}$. The (002) and (100) surfaces are flat at an atomic level, within which the $\overline{CN }$ of each Zn atoms at the same surface are the same, which are 7.50 and 6.80, respectively. Thus, a row of zinc atoms has been added on the atomically flat (002) and (100) crystal surfaces to tune the $\overline{CN }$ of surface Zn atoms, yielding uneven (002) and (100) surfaces, as shown in Fig. 6a. The $\overline{CN }$ of the upper Zn atoms at the uneven (002) surface dropped dramatically from 7.50 to 3.82. As shown in Figs. 6b, S10, and S11, the H adsorption energies of uneven Zn (002) surface are significantly lowered as compared to the original flat (002) surface, regardless of the top site or bridge site, confirming the correlation between $\overline{CN }$ and adsorption energy. The lowest adsorption energies even approximate to 0 eV. Therefore, it is the higher $\overline{CN }$ of atomically flat Zn (002) surface that delivers inert HER activity. Once the Zn (002) surface is uneven at an atomic level, the HER activity is no longer inert. Similarly, the upper Zn atoms at the reconstructed uneven (100) surface with lowered $\overline{CN }$ show lower $\Delta {G}_{{{\text{H}}}^{*}}$ (Figs. 6b and S10), which further confirming the relationship between $\overline{CN }$ and $\Delta {G}_{{{\text{H}}}^{*}}$. As a result, the $\overline{CN }$ of the surface Zn atom is proposed as a key descriptor of HER activity.

The surface Zn atoms with higher generalized coordination numbers would show lower HER activity. Zn (002) plane is more conducive to suppressing HER, and lots of efforts have already been made to achieve Zn anode with (002) crystal plane preferred exposed surface. On the other hand, considering that the edge sites would hold lower generalized coordination numbers, edge site should be minimized via increasing the particle size or additive design.

## Conclusions

In summary, HER activities on several crystal surfaces containing Zn (002), (100), (101), (102), and (103) surfaces are studied from both the thermodynamic and kinetic aspects in this work. The obtained results show that the adsorption free energy (∆*G*_H_) of a hydrogen atom adsorbed on Zn (002) and (100) surfaces are higher than that on Zn (101), (102), and (103) surfaces. The rate-limiting step of HER on the Zn (002) and (100) surfaces is the Volmer step, whereas the HER activity on the Zn (101), (102), and (103) surfaces is restricted by the Tafel step. The Zn (002) and (100) surfaces are flat at an atomic level in the used structural model, while the Zn (101), (102), and (103) surfaces are uneven surface at an atomic level. The atomically flat (002) and (100) surfaces would process higher generalized coordination numbers than uneven (101), (102), and (103) surfaces. The Zn atoms at a flat surface would process a higher coordination number. The mechanisms of the relatively weaker HER activity on the Zn (002) surface are revealed, which can be attributed to the higher $\overline{CN }$ of surface Zn atom. Once the surface of Zn (002) or Zn (100) slab modes is not flat at an atomic level, the uneven Zn (002) surface and Zn (100) surface would show significantly higher HER activity than the flat Zn (002) surface. The $\overline{CN }$ of the surface Zn atom is proposed as a key descriptor of HER activity after our comprehensive analysis. According to the key descriptor $\overline{CN }$, the most stable adsorption site for the H atom and the HER activity could be predicted. It is proposed that tuning the $\overline{CN }$ of the surface Zn atom would be a vital strategy to inhibit HER on the Zn anode. From a visualized perspective, the exposed surface of Zn anode needs to be flat at an atomic level to avoid HER at the surface. This work provides a theoretical understanding of HER on the Zn surface and a guideline to suppress HER on the Zn anode.

## Supplementary Information

Below is the link to the electronic supplementary material.

Supplementary file1 (Docx 12,101 KB)
