# Supplementary material for: Fundamental Understanding of Hydrogen Evolution Reaction on Zinc Anode Surface: A First-Principles Study
Source: Nanomicro Lett. 2024 Feb 6;16:111. doi: 10.1007/s40820-024-01337-0 (PMC11250978; doi:10.1007/s40820-024-01337-0)
Supplement: Supplementary file 1 — Supplementary file1 (Docx 12,101 KB) [file 40820_2024_1337_MOESM1_ESM.docx]

Support Information for

**The Fundamental Understanding of Hydrogen Evolution Reaction on Zinc Anode Surface: A First-Principles Study**

Xiaoyu Liu^1^, Yiming Guo^1^, Fanghua Ning^1,^ *, Yuyu Liu^1^, Siqi Shi^2^, Qian Li^3^, Jiujun Zhang^1^, Shigang Lu^1^, and Jin Yi^1,^ *

^1^ Institute for Sustainable Energy & Department of Chemistry, Shanghai University, Shanghai 200444, China.

^2^ School of Materials Science and Engineering, Shanghai University, Shanghai 200444, China.

^3^ College of Materials Science and Engineering, National Engineering Research Center for Magnesium Alloys, Chongqing University, Chongqing 400044, China.

*Corresponding author

E-mail: fhning@shu.edu.cn; jin.yi@shu.edu.cn

**Supplementary Figures**


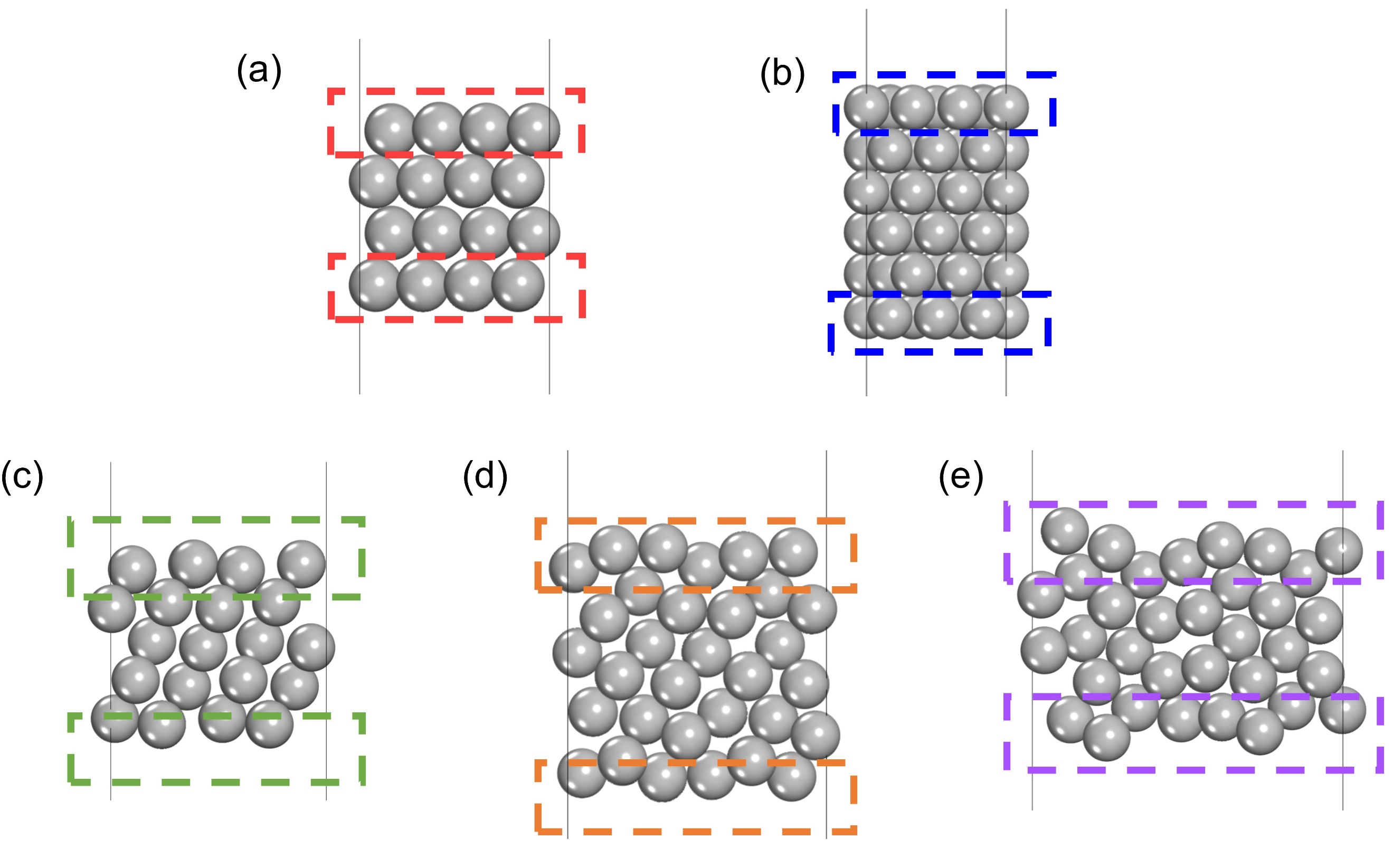


**Fig. S1** The side views of the slab models. **a** Zn (002) surface, **b** Zn (100) surface, **c** Zn (101) surface, **d** Zn (102) surface, **e** Zn (103) surface.


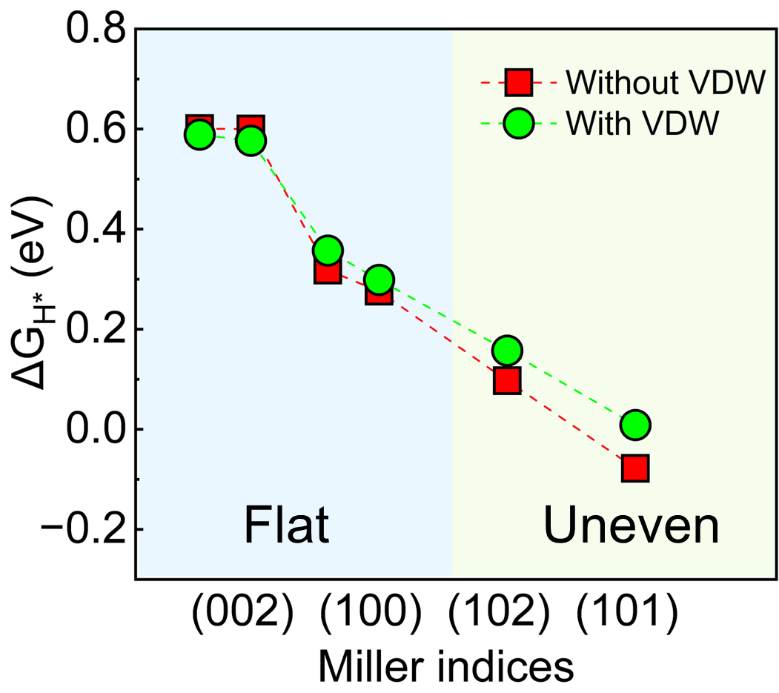


**Fig. S2** The $\Delta G_{H^{*}}$ obtained after considering Van der Waals (VDW) interactions.


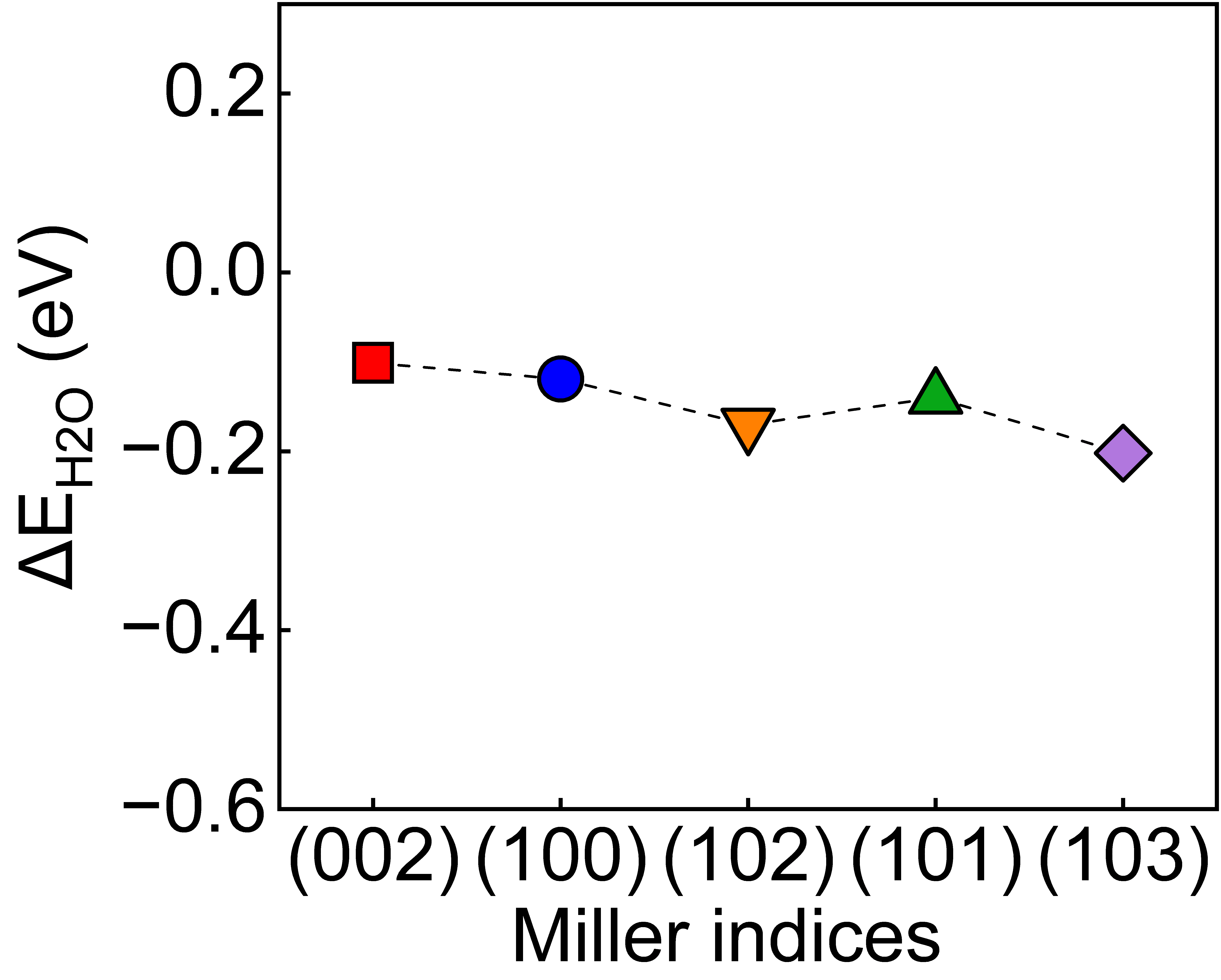


**Fig. S3** The comparative water adsorption energy on several crystal surface of Zn anode.


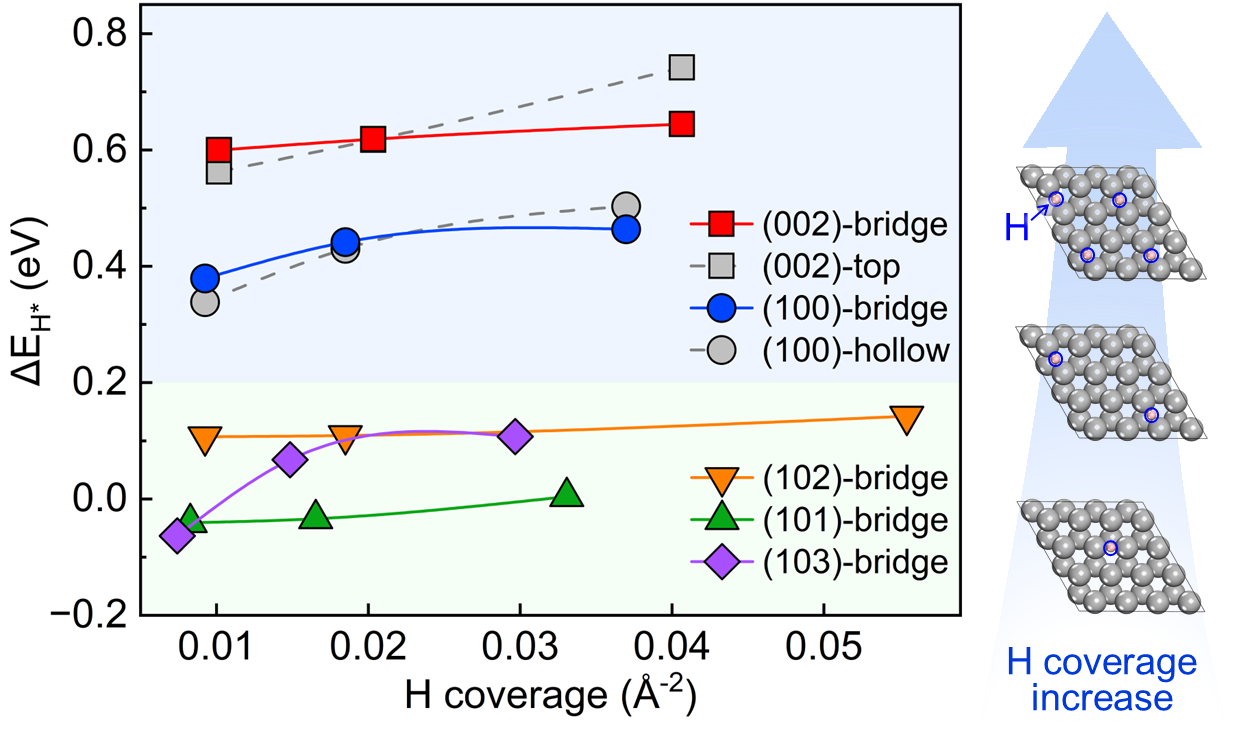


**Fig. S4** Hydrogen adsorption energy ($\Delta E_{H^{*}}$) at several crystal surfaces of the zinc anode as a function of different hydrogen coverage. The H coverage indicates the number of hydrogens absorbed per unit area.


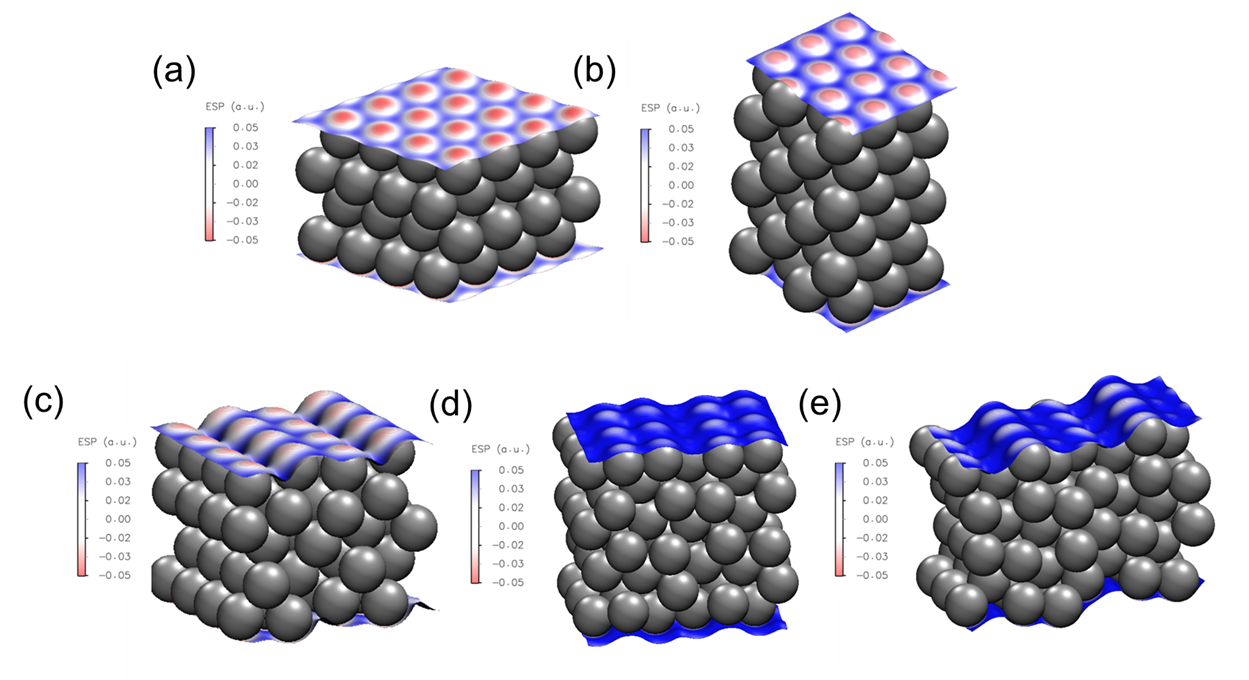


**Fig. S5** The electrostatic potential at several crystal surface of Zn anode. **a** Zn (002) surface, **b** Zn (100) surface, **c** Zn (101) surface, **d** Zn (102) surface, e Zn (103) surface.


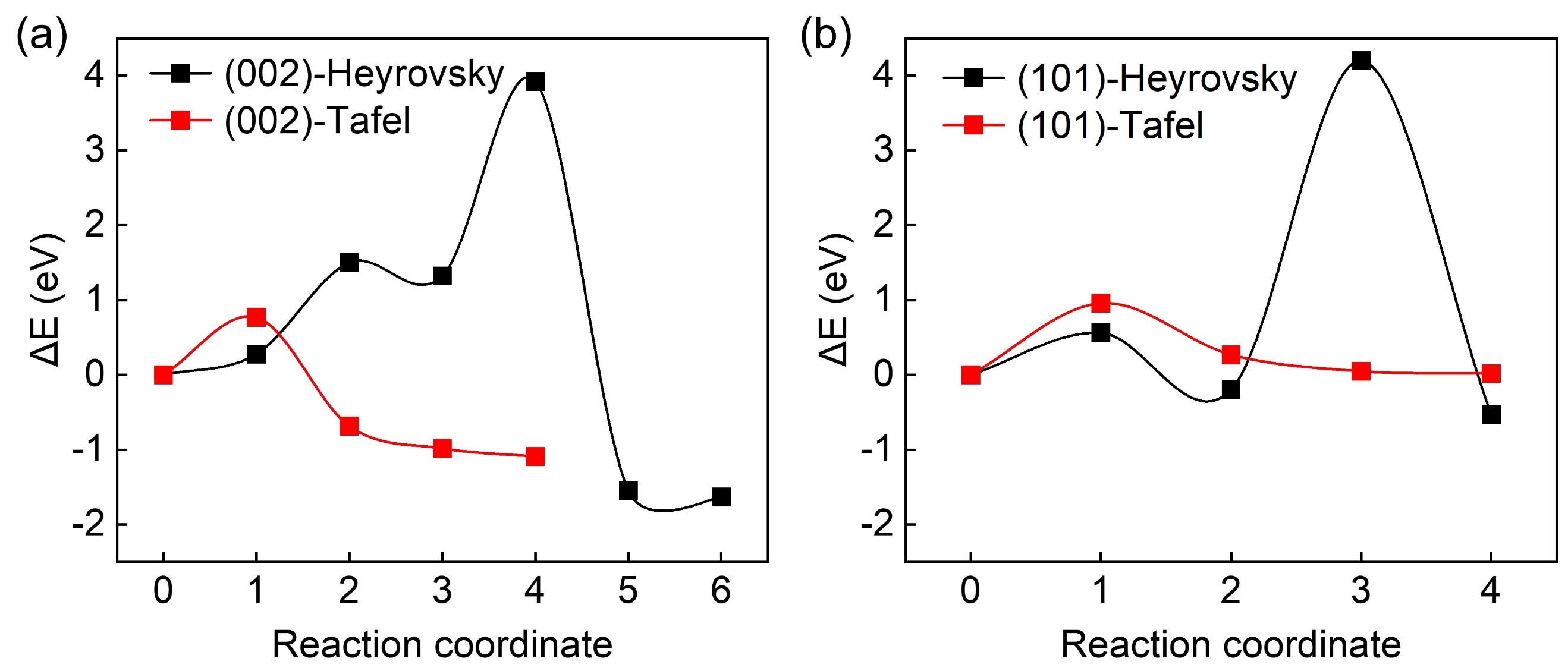


**Fig. S6** The energy barriers and reaction pathways of Heryrovsky step and Tafel step on **a** flat crystal surface (002) and **b** uneven crystal surface (101) of Zn anode.


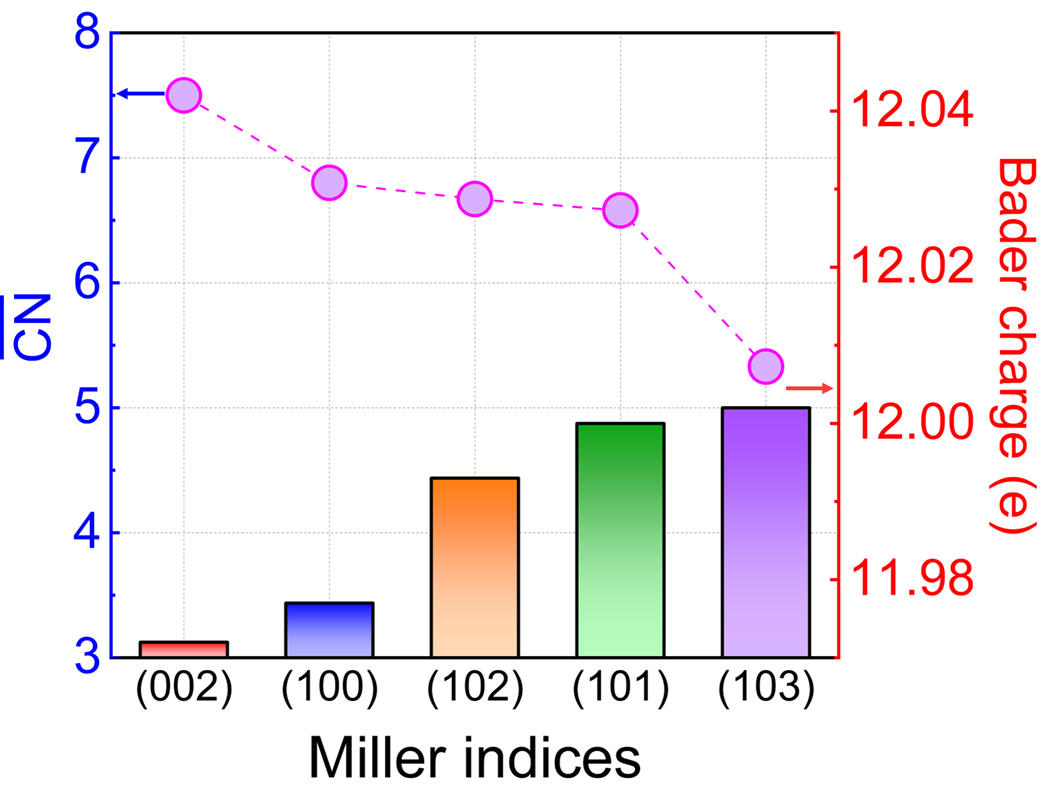


**Fig. S7** The Bader charge of surface Zn, and correlation between the Bader charge and generalized coordination number $\bar{CN}$.
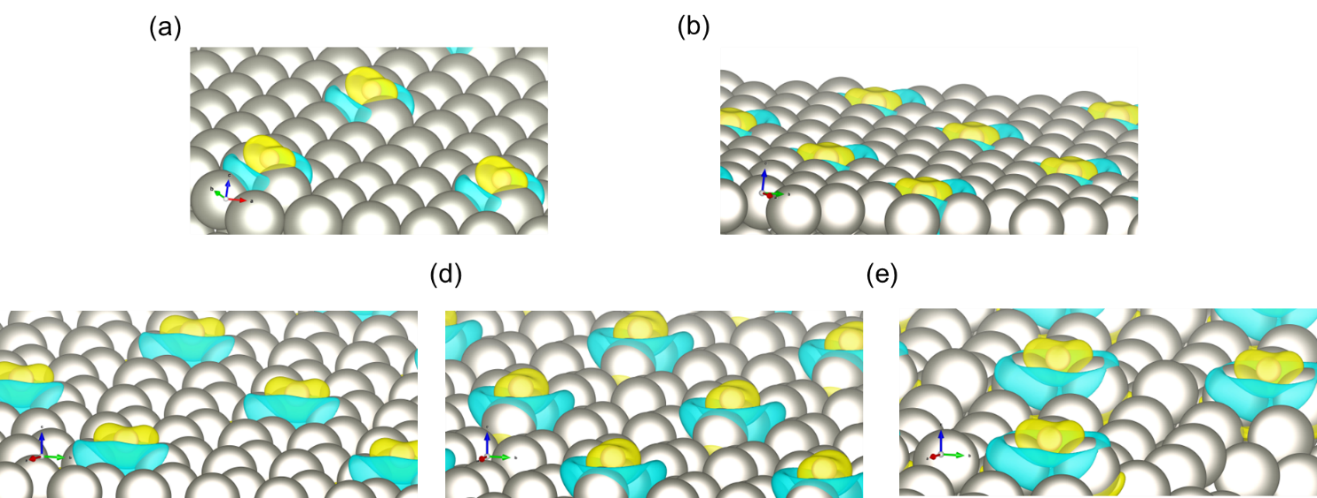


**Fig. S8** The differential charge density induced by H adsorption for **a** Zn (002), **b** Zn (100), **c** Zn (101), **d** Zn (102), **e** Zn (103) surface models. The isosurfaces are all set to 0.001 e/Bohr^3^. The yellow and blue isosurfaces indicate loss and gain electrons, respectively.


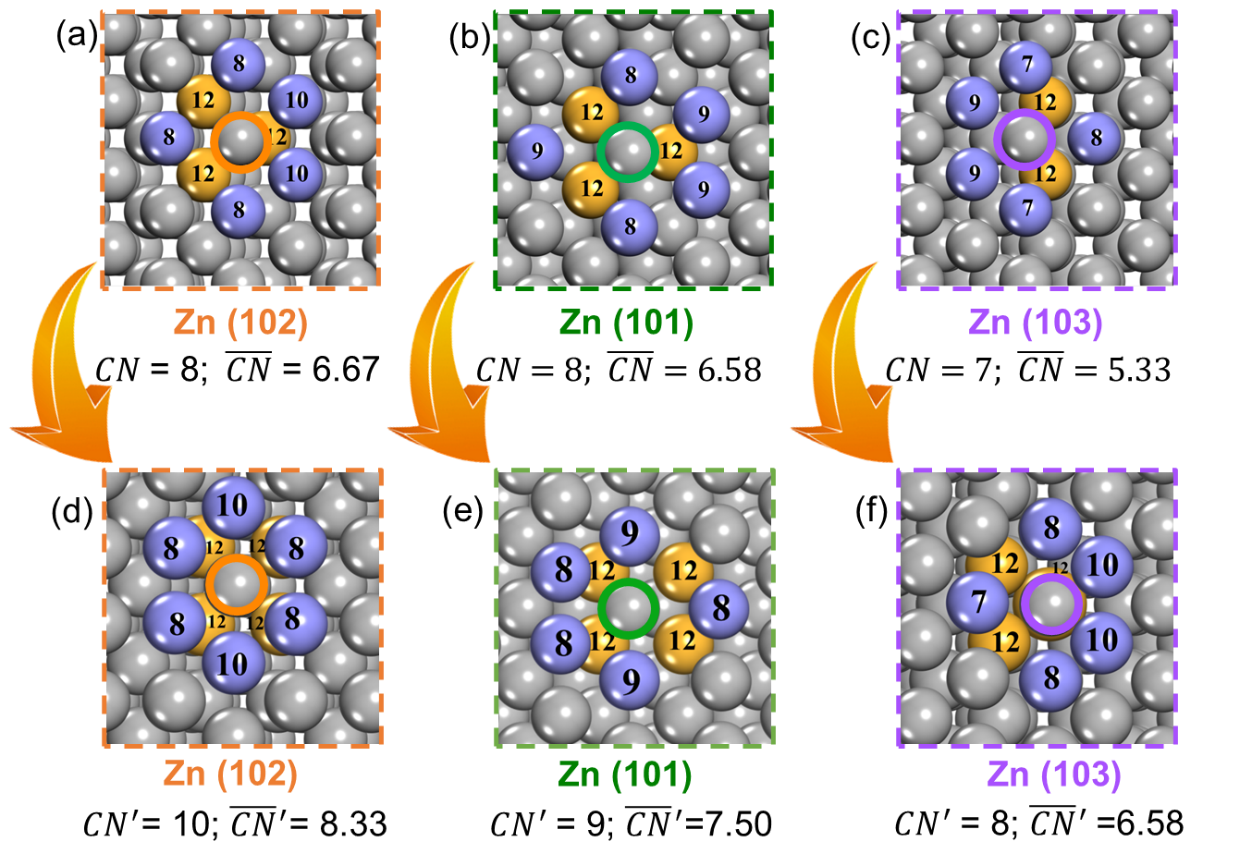


**Fig. S9** The schematic diagram of the $\bar{CN}$of Zn atom with lowest $\Delta G_{H^{*}}$ at **a** Zn (102), **b** Zn (101), and **c** Zn (103) surface. The schematic diagram of the$\bar{CN}^{'}$of Zn atom with higher $\Delta G_{H^{*}}$ at **d** Zn (102), **e** Zn (101), and **f** Zn (103) surface.


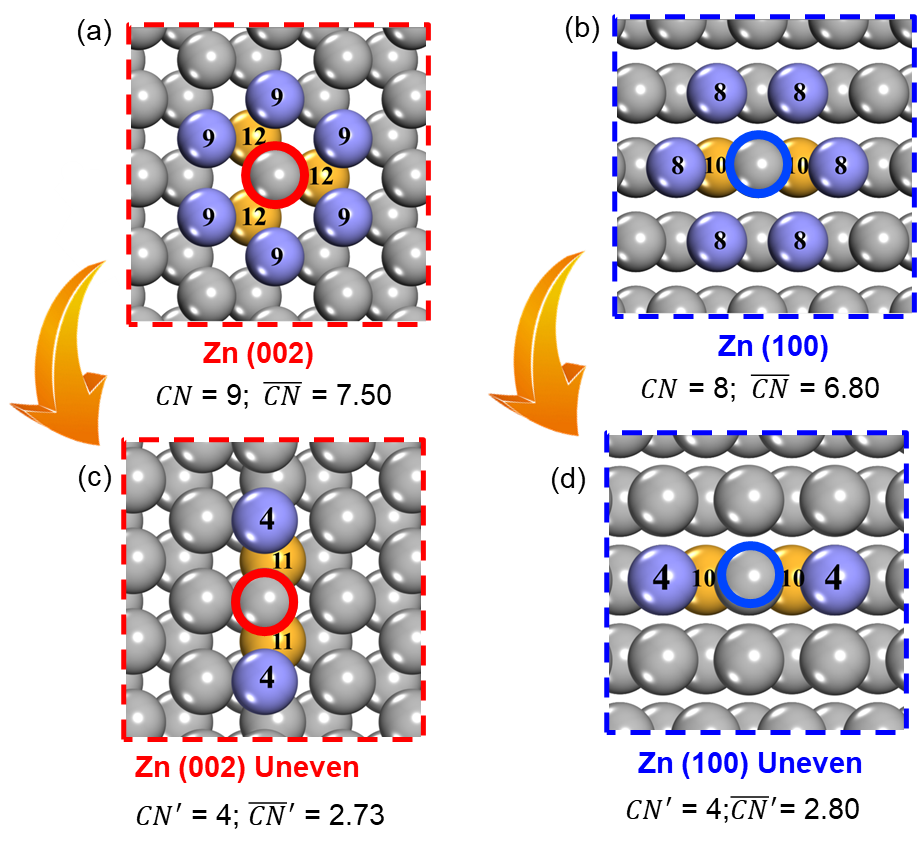


**Fig. S10** The schematic diagram of the $\bar{CN}$of Zn atom at **a** Zn (002) and **b** Zn (100) surface. The schematic diagram of $\bar{CN}^{'}$of Zn atom at **c** uneven Zn (002) and **d** uneven Zn (100) surface.


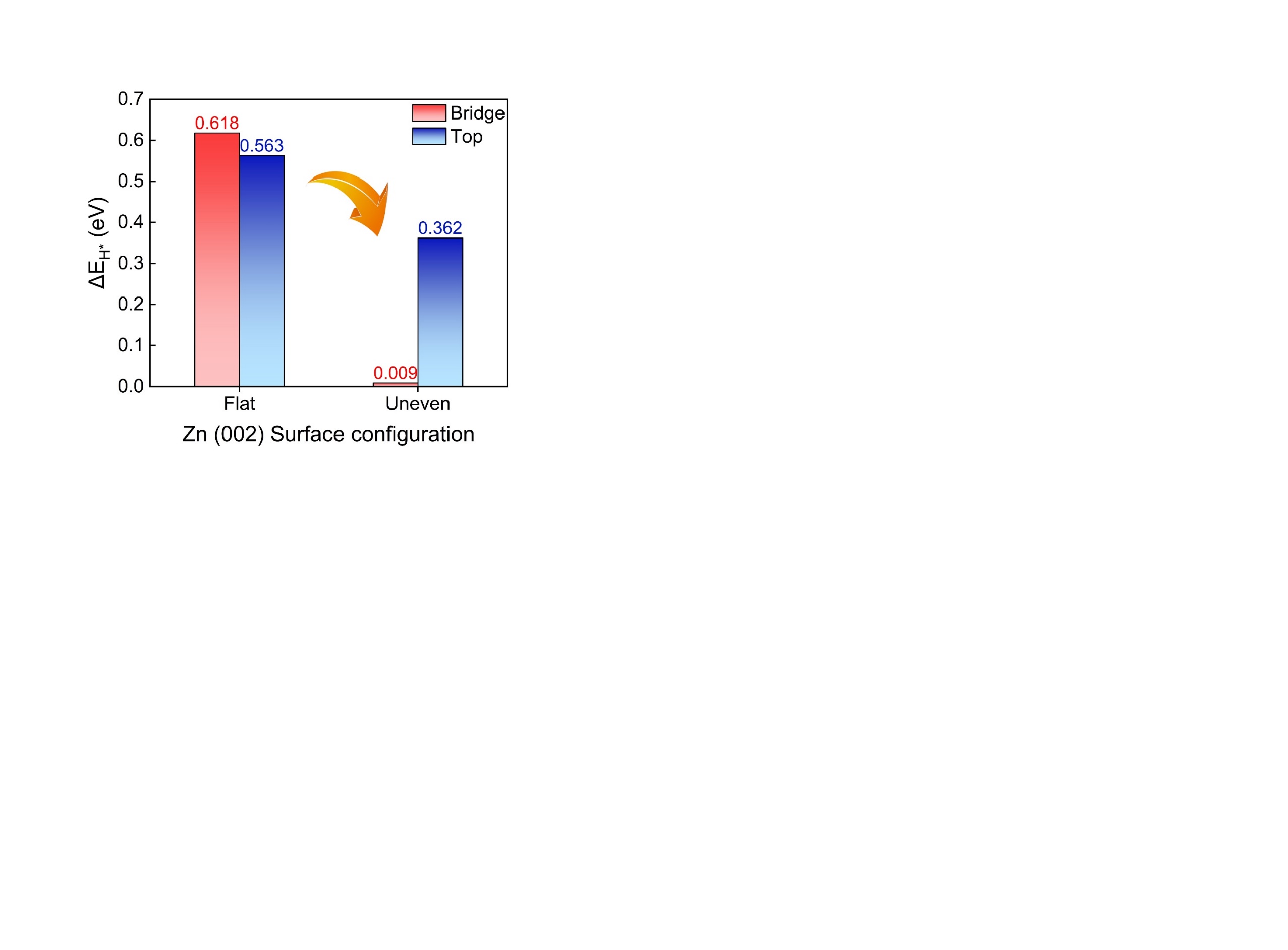


**Fig. S11** The adsorption energy of H on flat and uneven Zn (002) surface.

**Supplementary Tables**

**Table S1.** The computational details about k-points densities applied for Brillouin zone integration and the information of slab models.

| Miller indices | Lattice parameters | Layer number | Atom number | K-Points | k-points densities (/Å^-1^) |
| --- | --- | --- | --- | --- | --- |
| (0 0 2) | a = b = 10.66 Å  c = 39.78 Å  α = β = 90.00°  γ =120.00° | 4 | 64 | 3 3 1 | 32 32 40 |
| (1 0 0) | a = 8.01 Å  b = 9.79 Å  c = 48.53 Å  α=β=γ= 90.00° | 6 | 72 | 3 3 1 | 24 29 49 |
| (1 0 1) | a = 11.87 Å  b = 10.44 Å  c = 37.00 Å  α = β = 90.00°  γ =102.70° | 5 | 80 | 3 3 1 | 36 31 37 |
| (1 0 2) | a = 13.53 Å  b = 7.99 Å  c = 29.69 Å  α = β =γ= 90.00° | 6 | 96 | 3 4 1 | 41 32 30 |
| (1 0 3) | a = 17.11 Å  b = 7.98 Å  c = 26.71 Å  α = β = 90.00°  γ =98.94° | 5 | 102 | 1 3 1 | 17 24 27 |

**Table S2.** The $\Delta G_{H^{*}}$ values and the corresponding structures for H adsorbed at different sites of Zn (101) surface.

| Surface structures | Adsorption site | $\Delta G_{H^{*}}$ (eV) |
| --- | --- | --- |
| 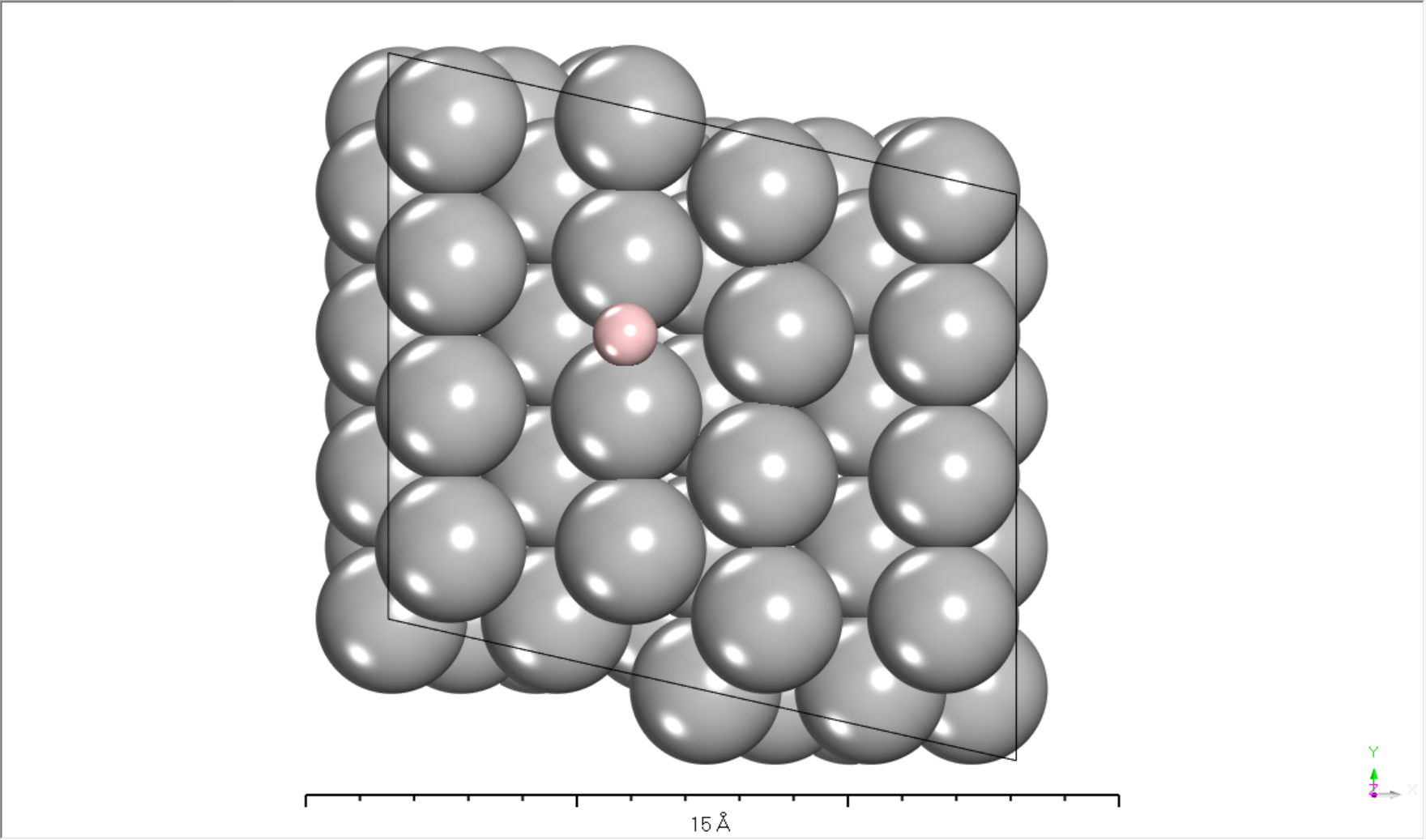 | Bridge | 0.023 |
| 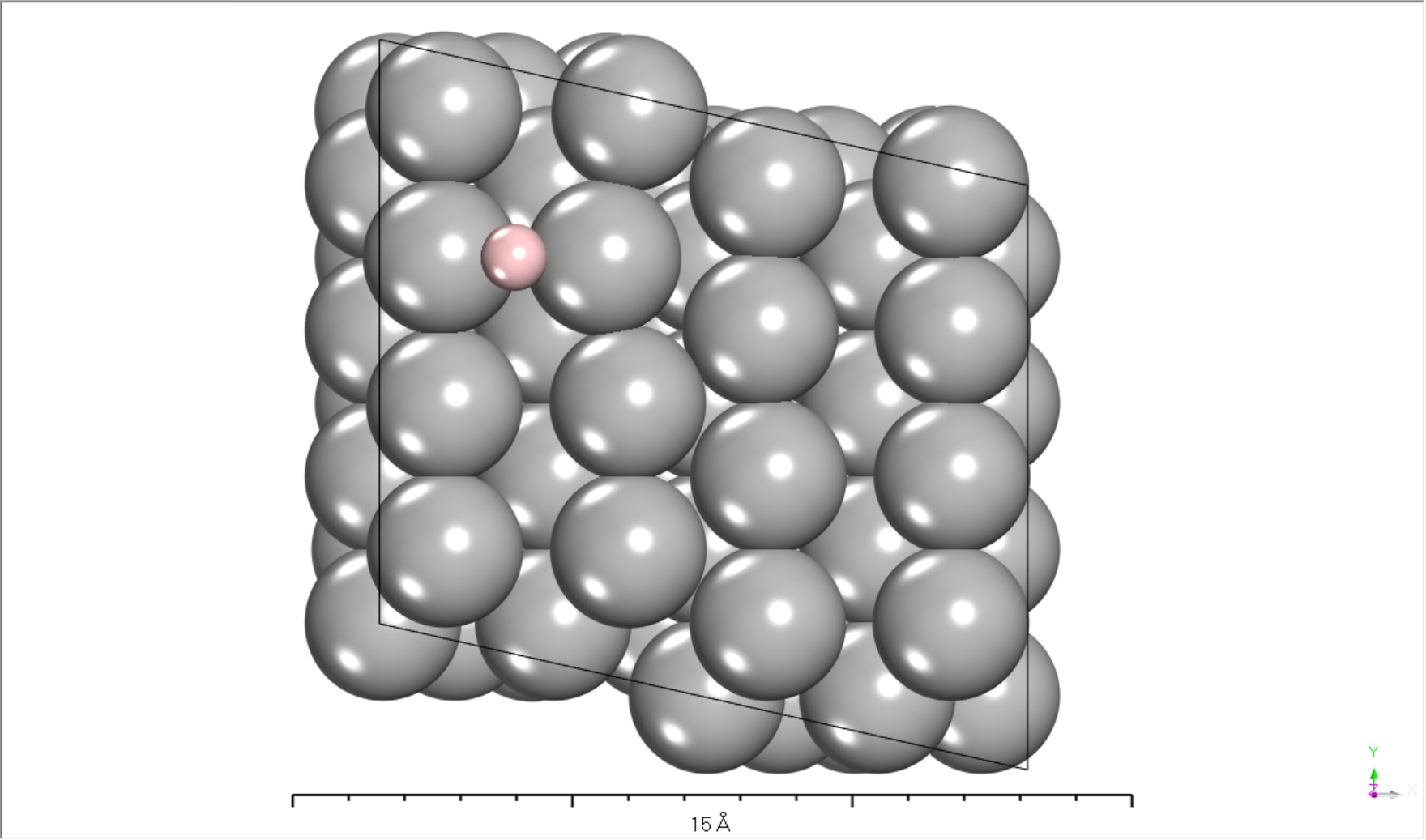 | Bridge | 0.363 |
| 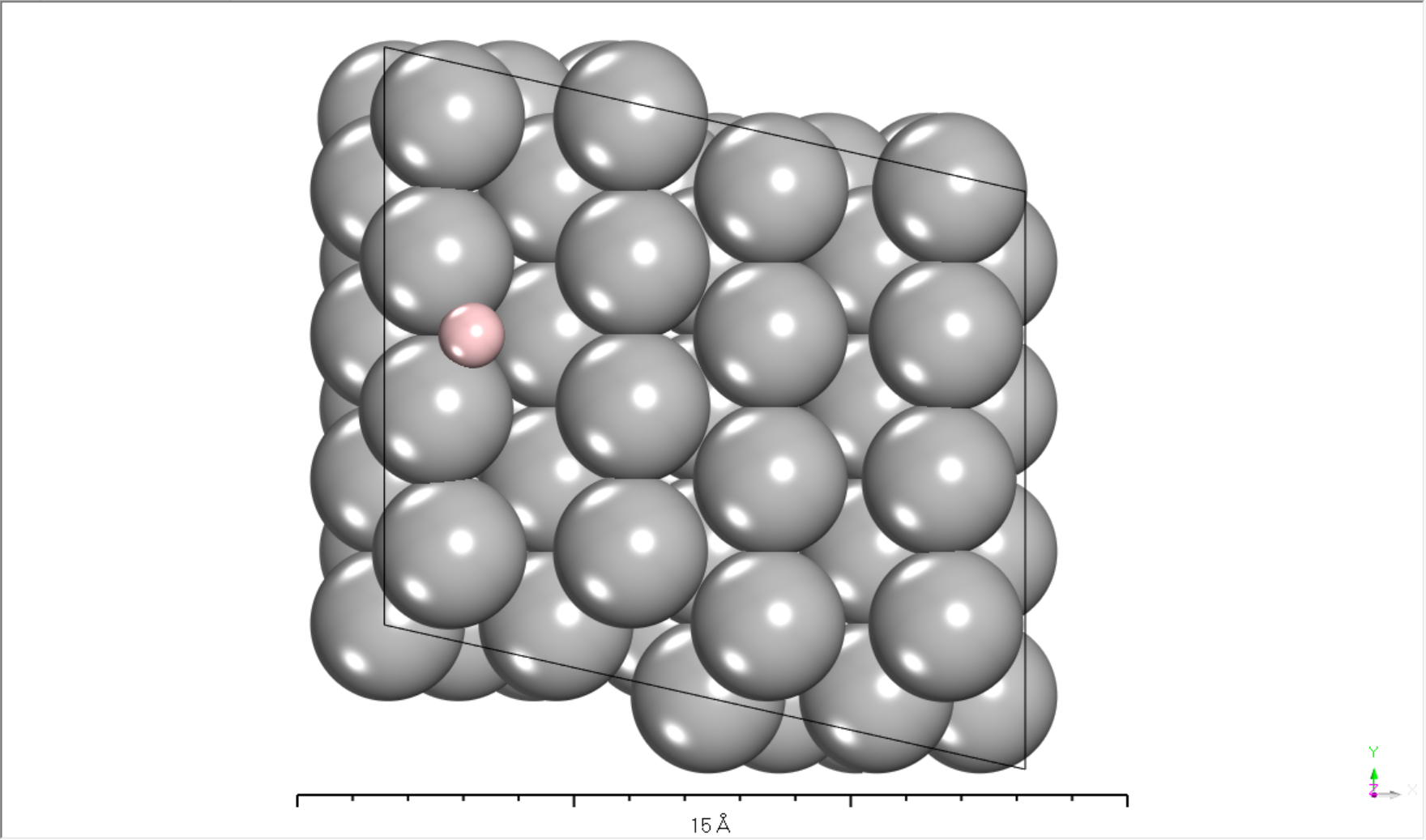 | Bridge | 0.250 |
| 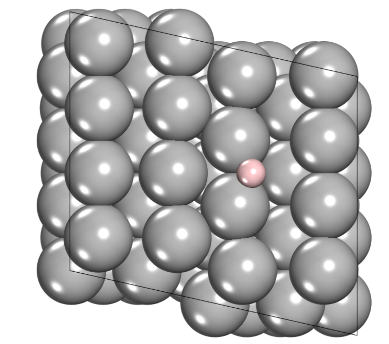 | Bridge | 0.242 |

**Table S3.** The $\Delta G_{H^{*}}$ values and the corresponding structures for H adsorbed at different sites of Zn (102) surface.

| Surface structures | Adsorption site | $\Delta G_{H^{*}}$ (eV) |
| --- | --- | --- |
| 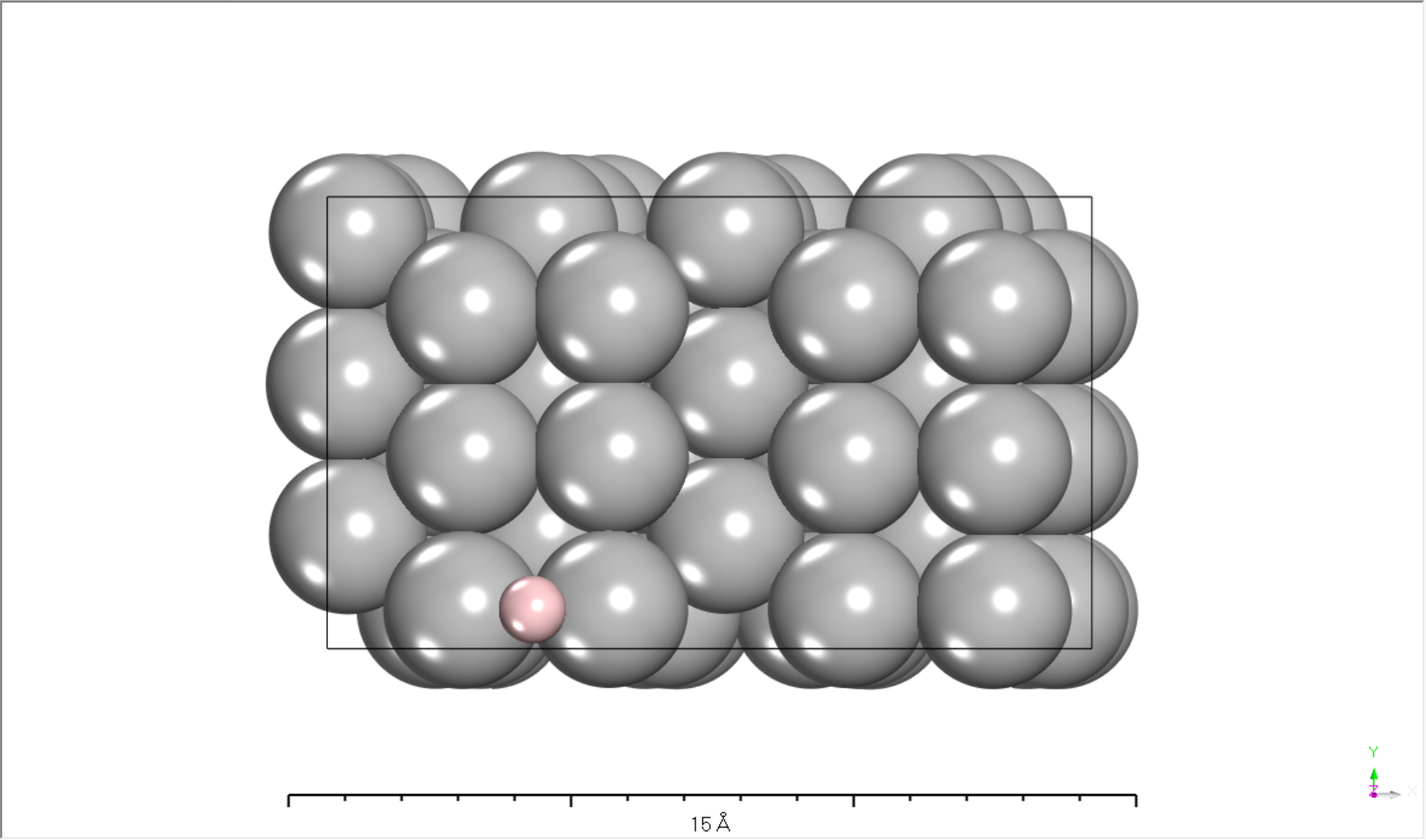 | Bridge | 0.174 |
| 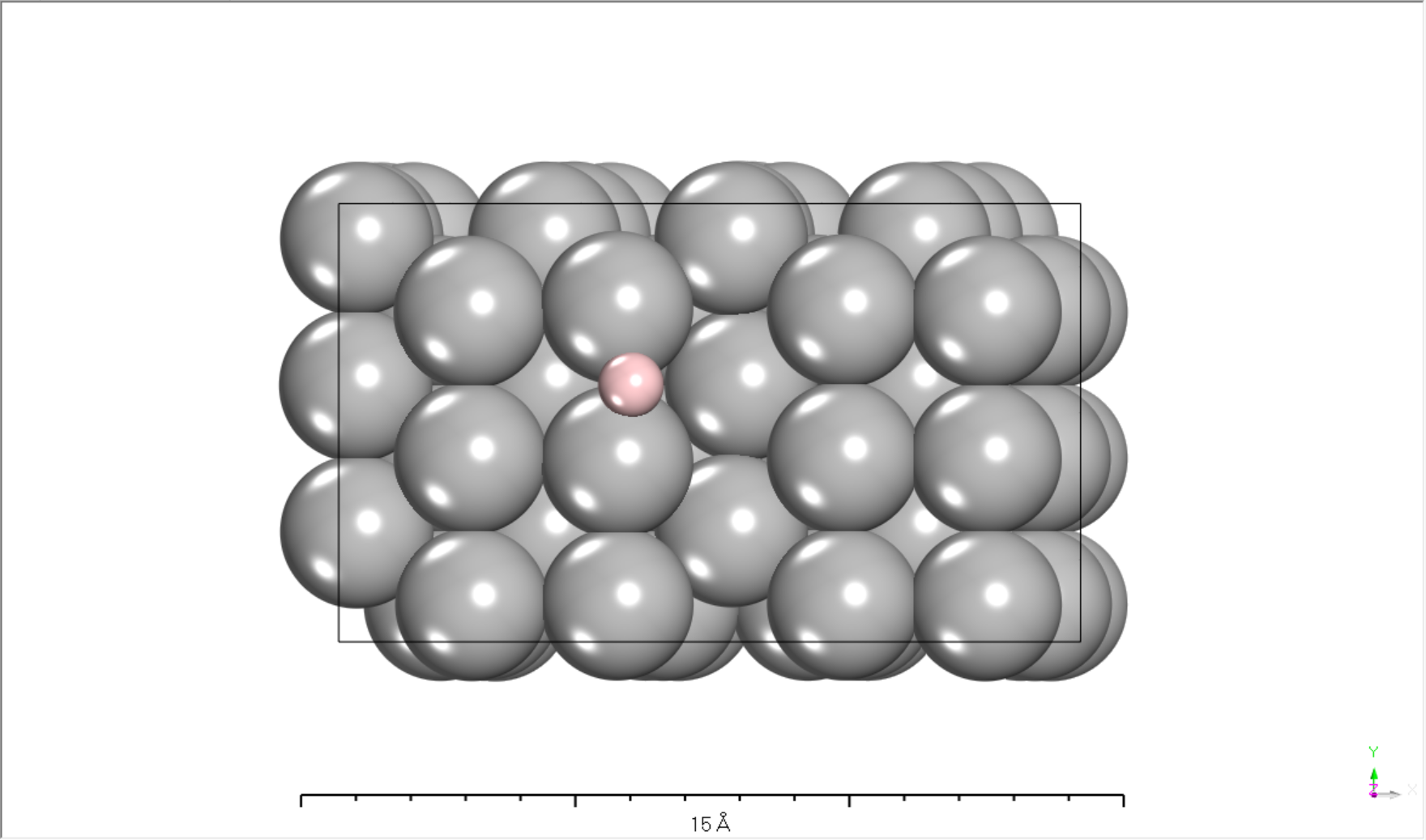 | Bridge | 0.334 |
| 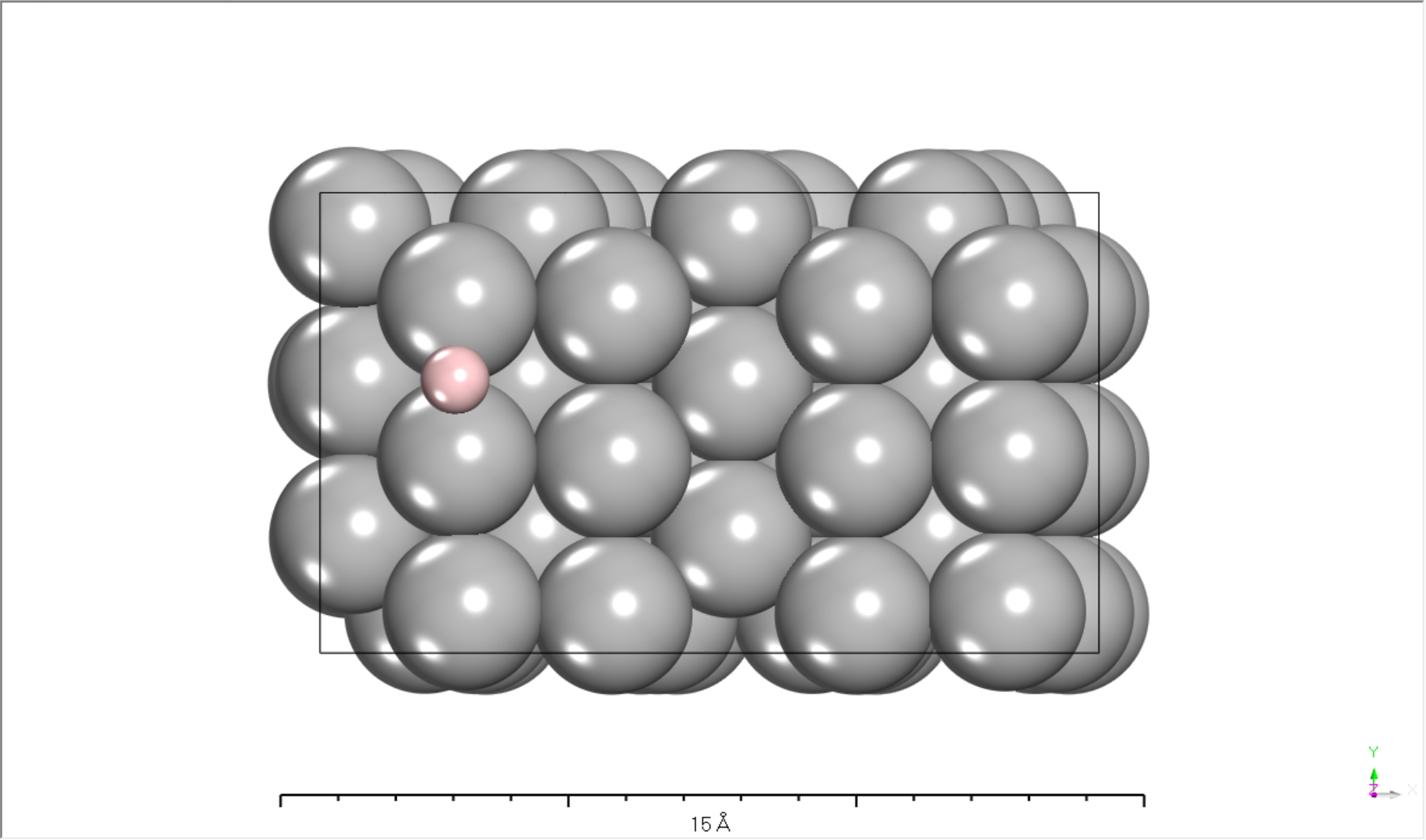 | Bridge | 0.277 |
| 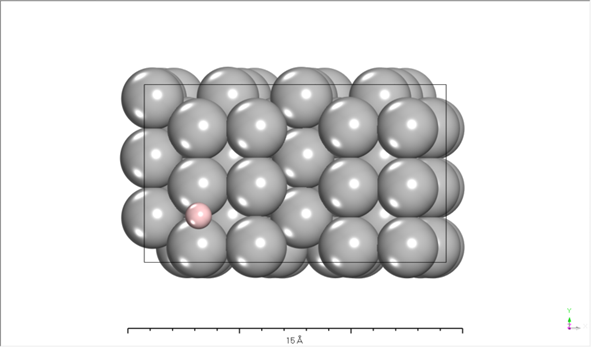 | Bridge | 0.285 |
| 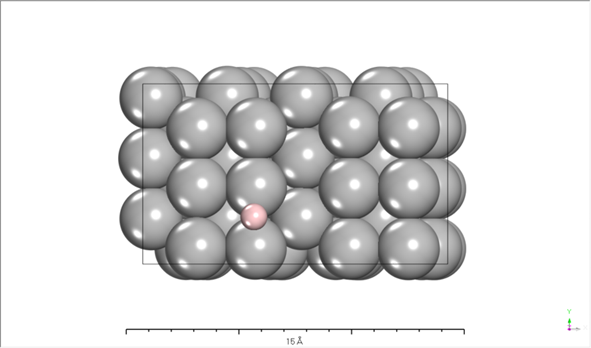 | Bridge | 0.328 |

**Table S4.** The $\Delta G_{H^{*}}$ values and the corresponding structures for H adsorbed at different sites of Zn (103) surface.

| Surface structures | Adsorption site | $\Delta G_{H^{*}}$ (eV) |
| --- | --- | --- |
| 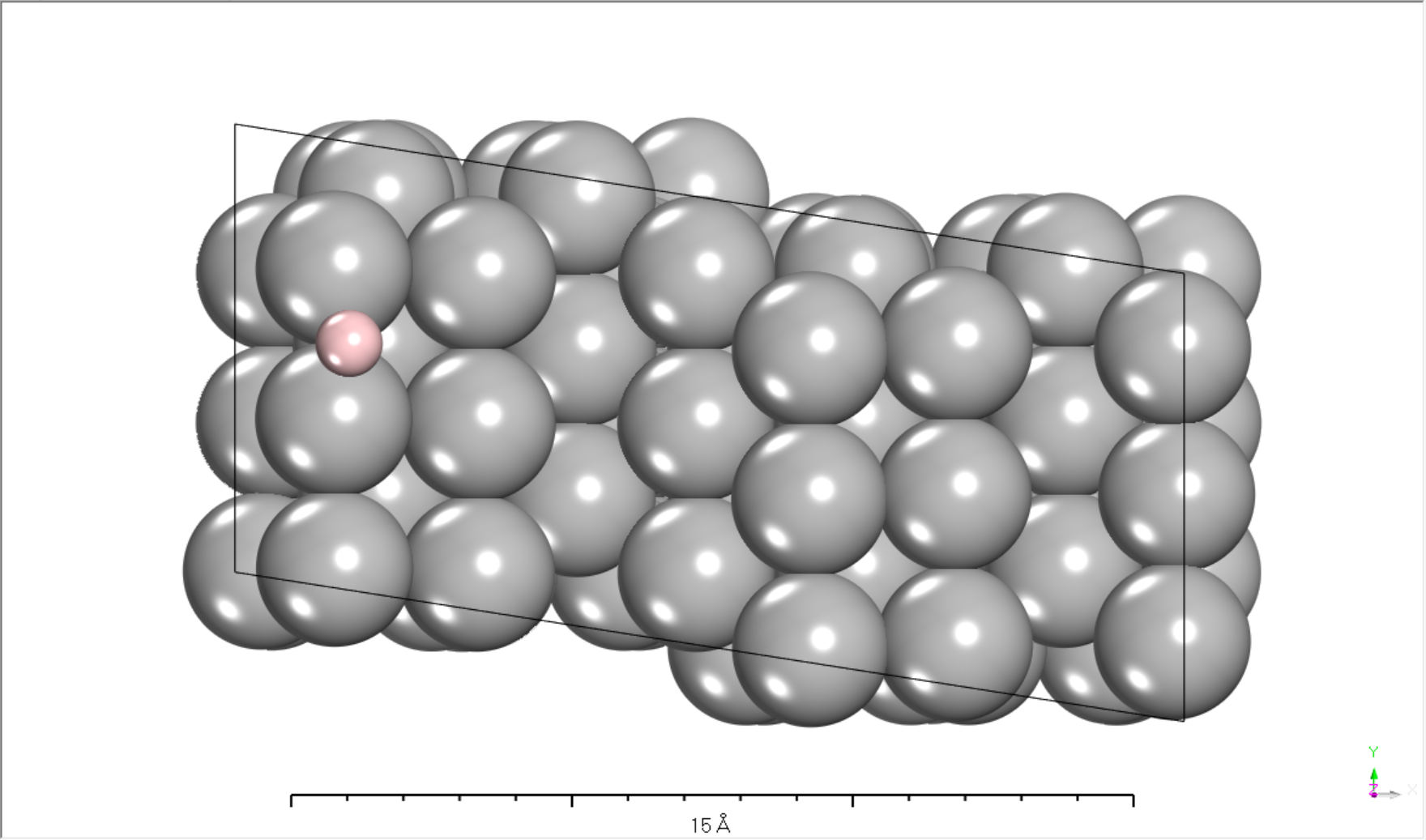 | Bridge | -0.054 |
| 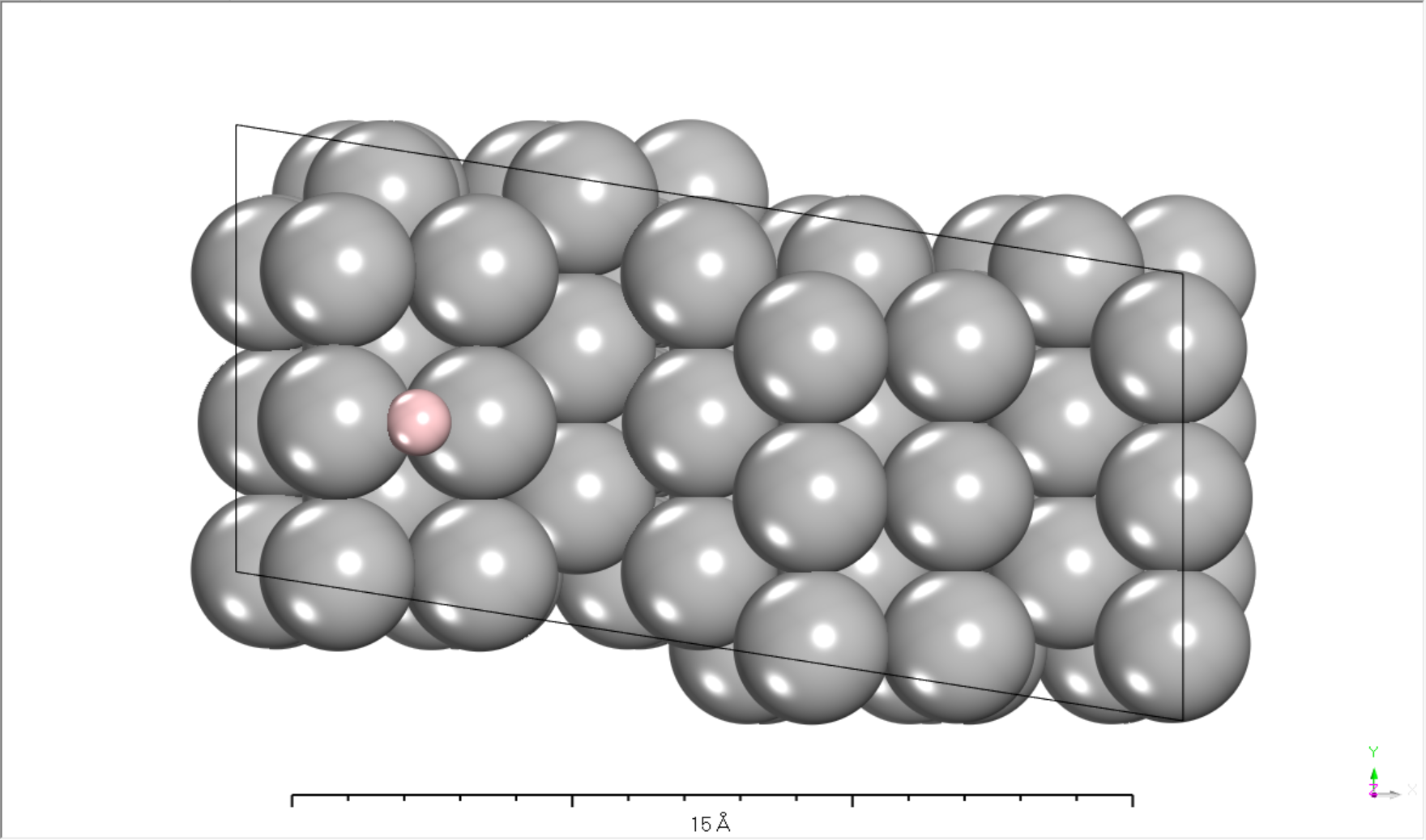 | Bridge | 0.058 |
| 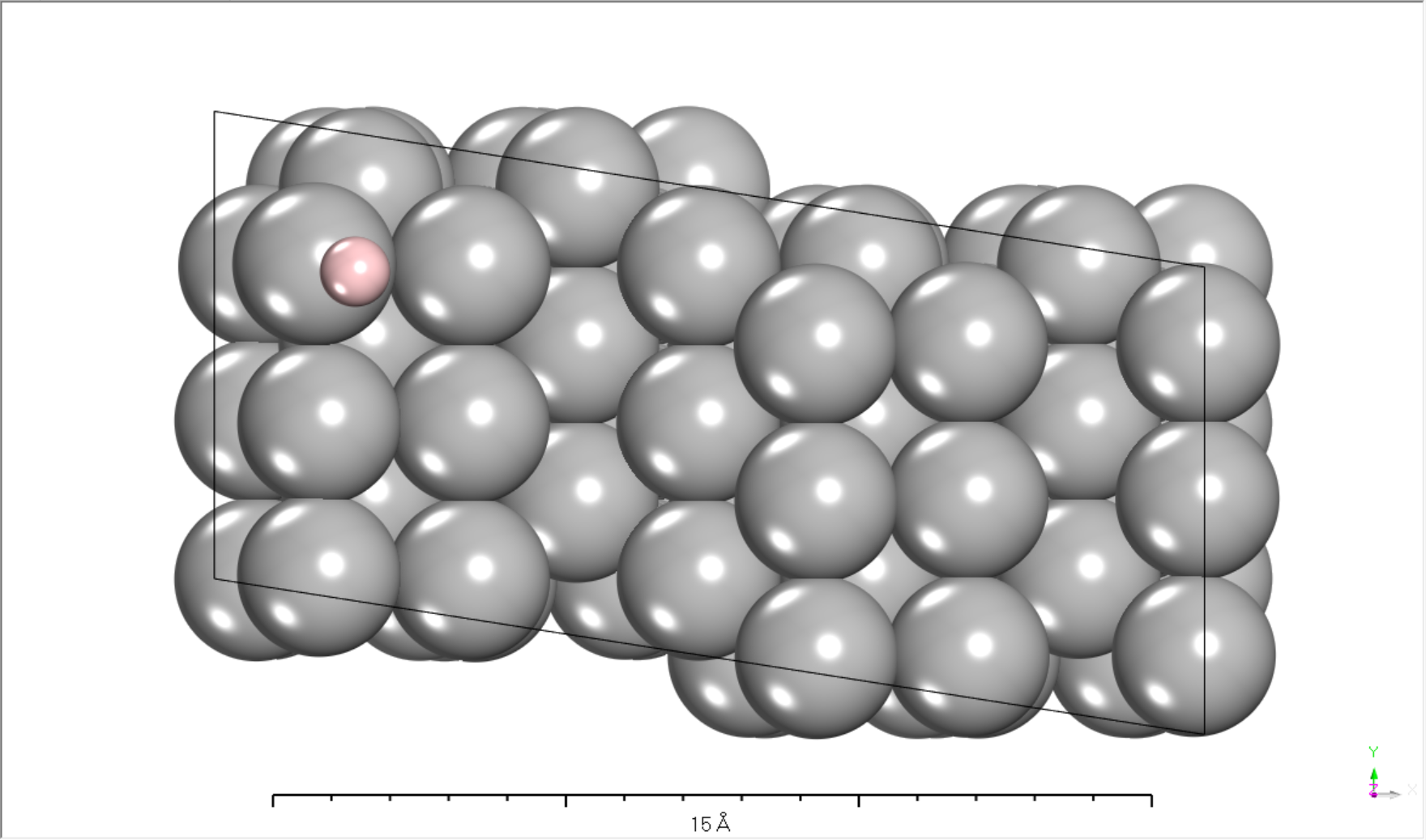 | Top | 0.199 |
| 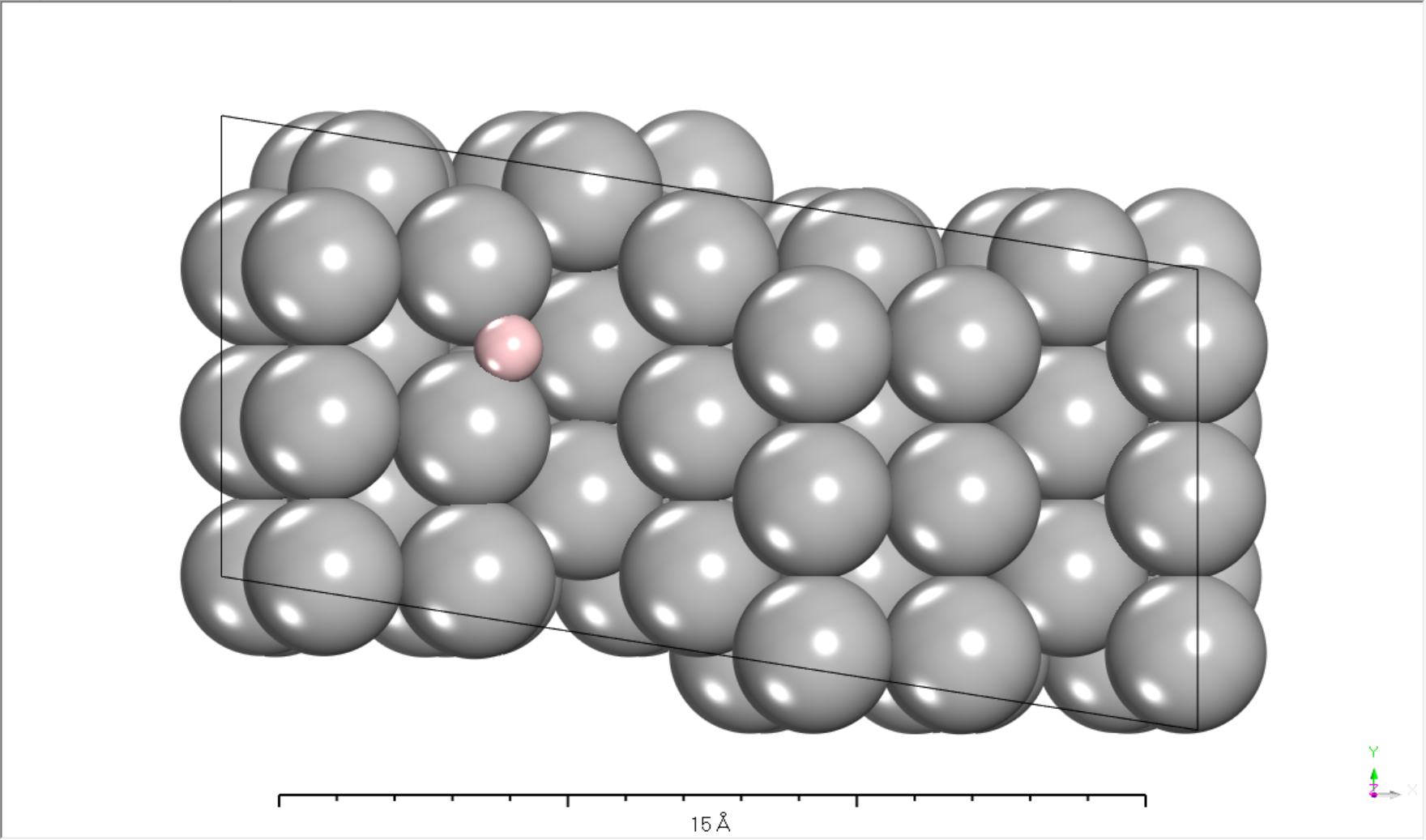 | Bridge | 0.340 |
|  |  |  |

**Table S5.** The $\Delta G_{H^{*}}$ values and the corresponding structures for H adsorbed at different sites of Zn (002) surface.

| Surface structures | Adsorption site | $\Delta G_{H^{*}}$ (eV) |
| --- | --- | --- |
| 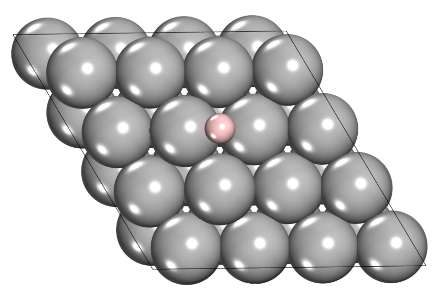 | Bridge | 0.589 |
| 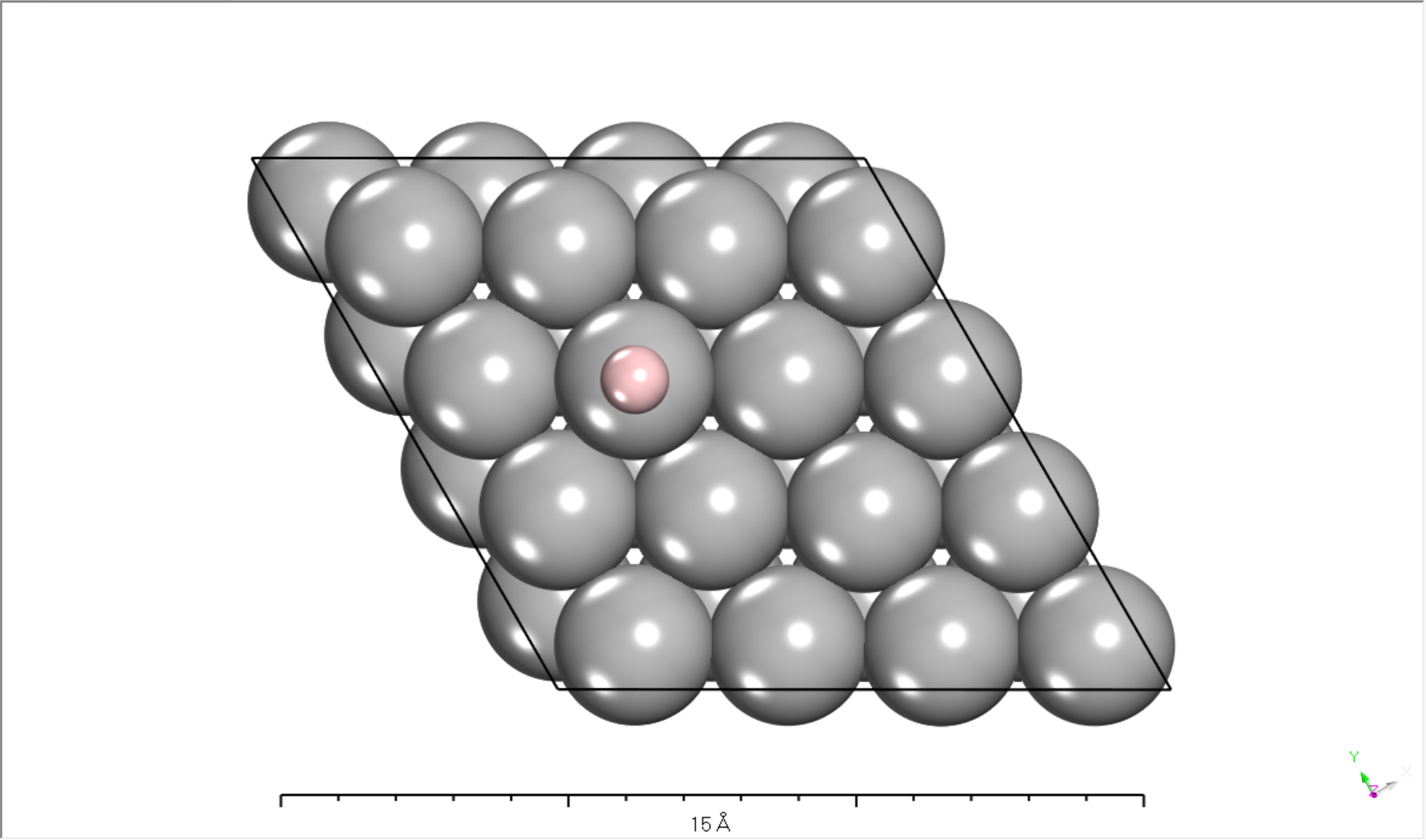 | Top | 0.577 |
| 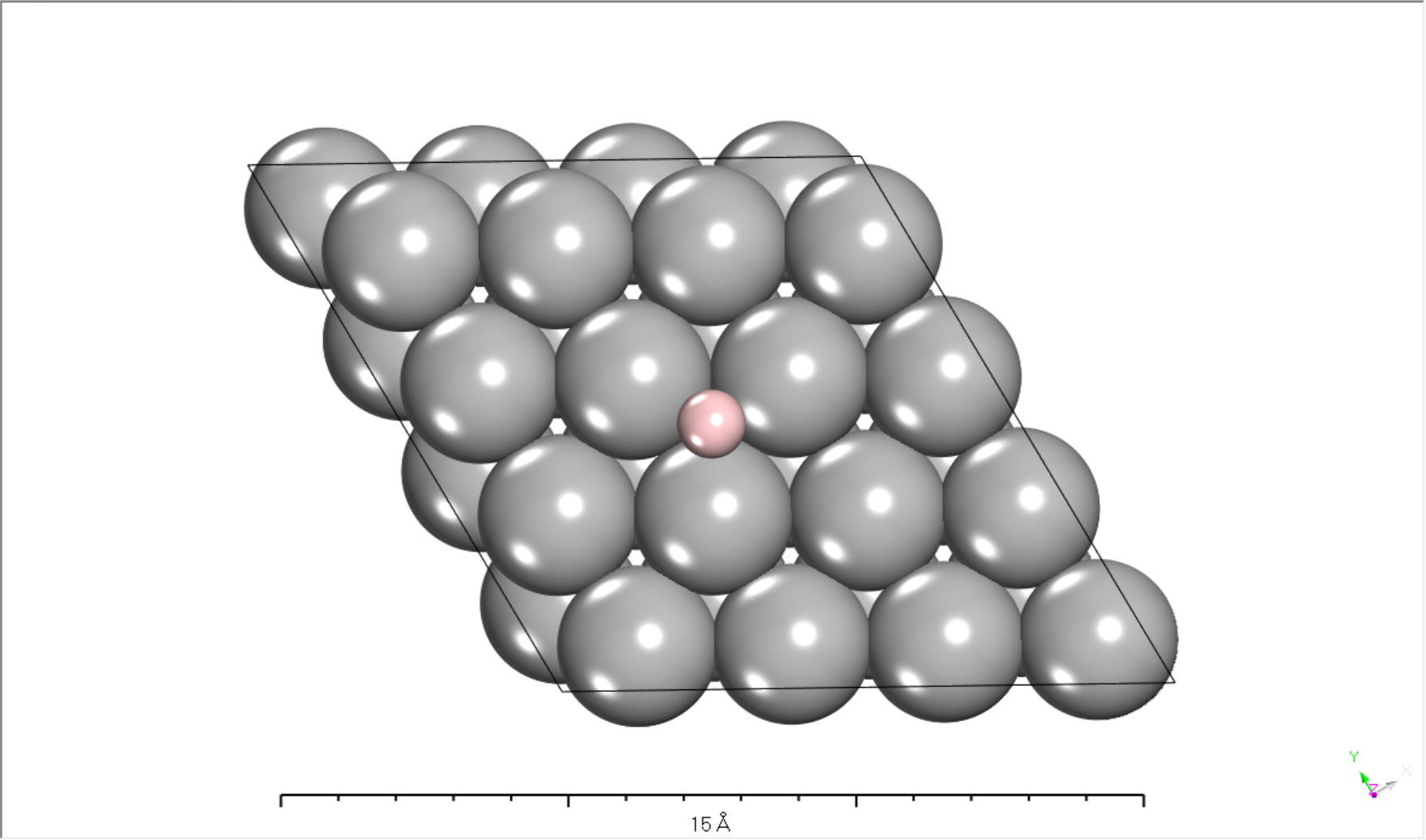 | Hollow-fcc | 0.647 |
| 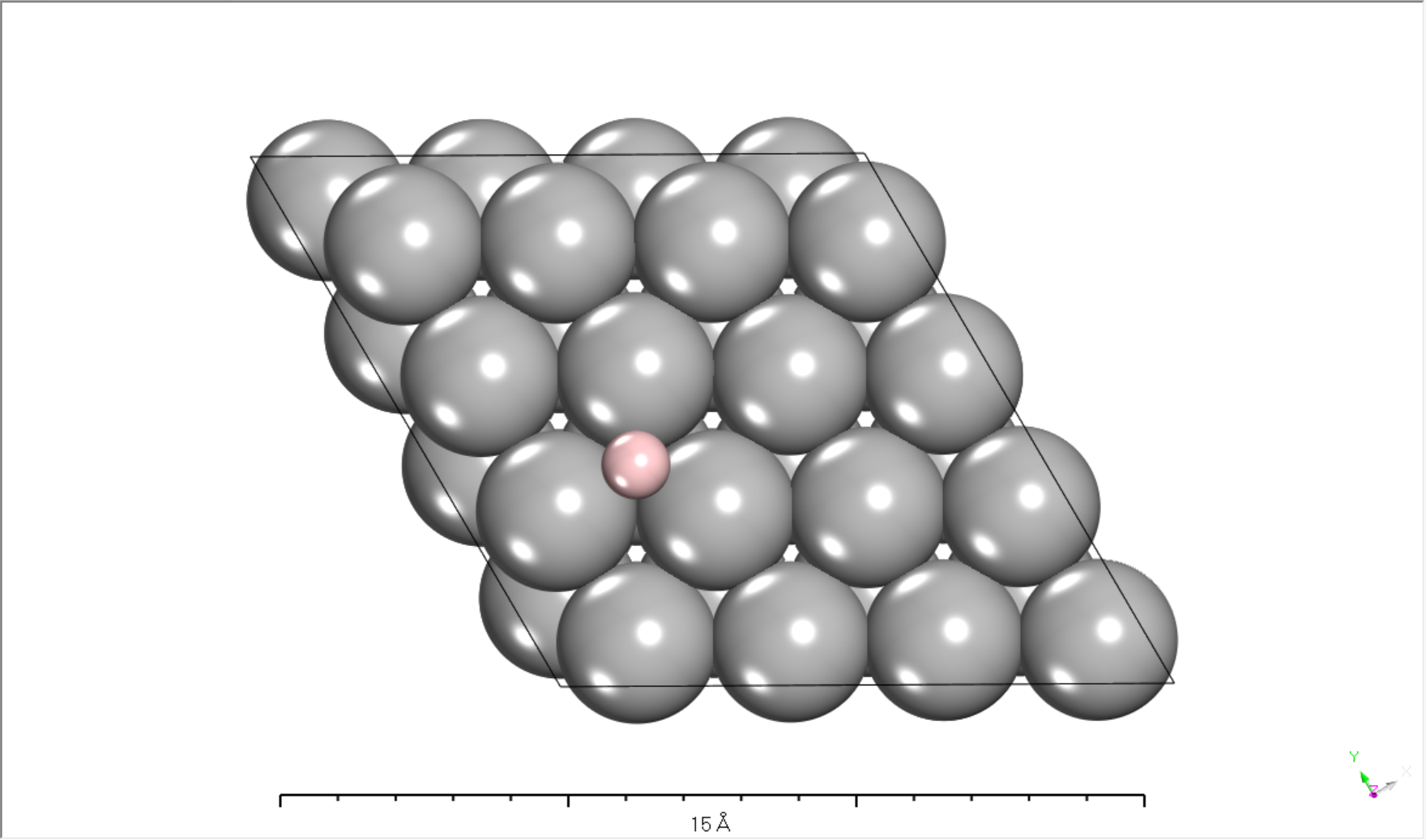 | Hollow-hcp | 0.630 |

**Table S6.** The $\Delta G_{H^{*}}$ values and the corresponding structures for H adsorbed at different sites of Zn (100) surface.

| Surface structures | Adsorption site | $\Delta G_{H^{*}}$ (eV) |
| --- | --- | --- |
| 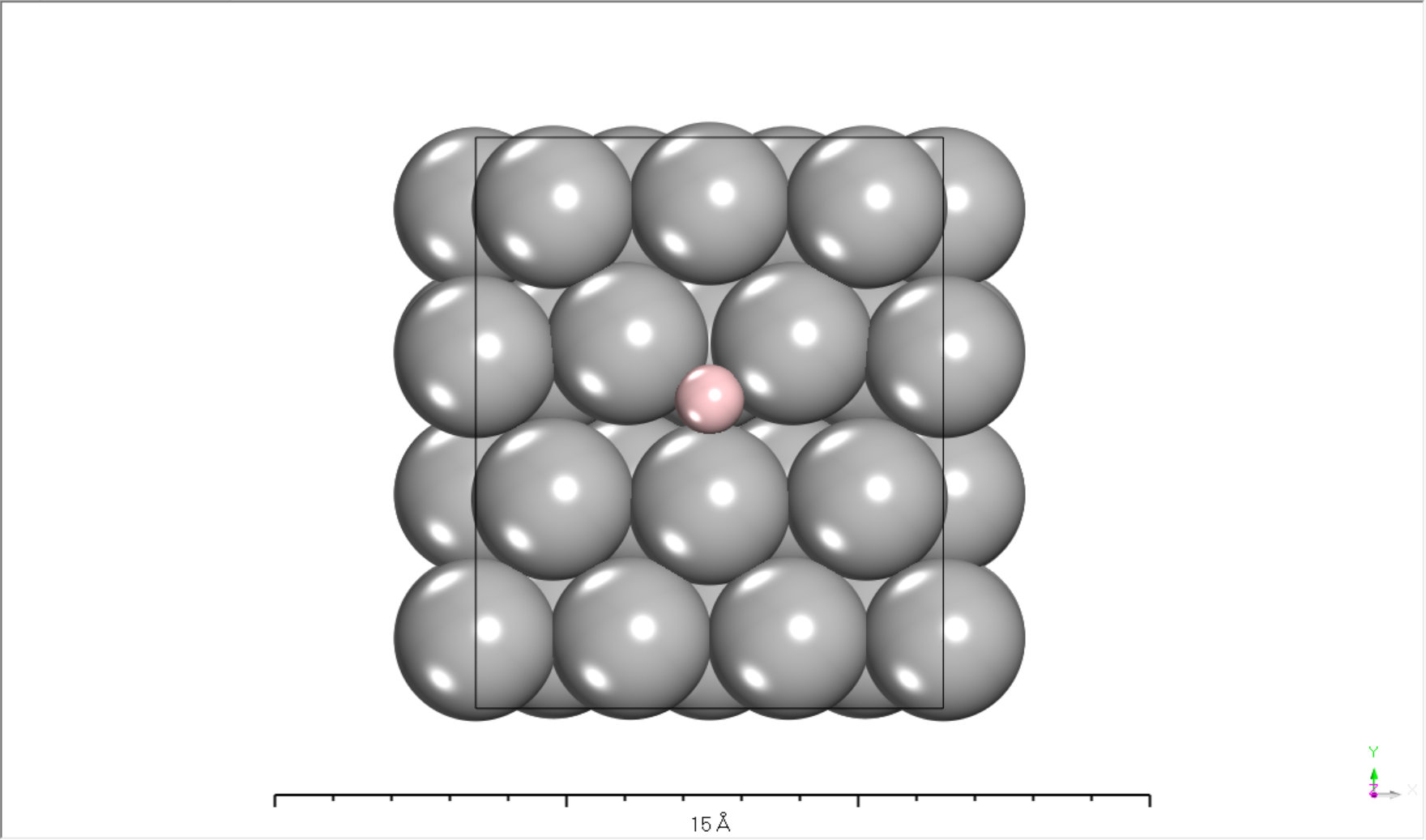 | Hollow | 0.290 |
| 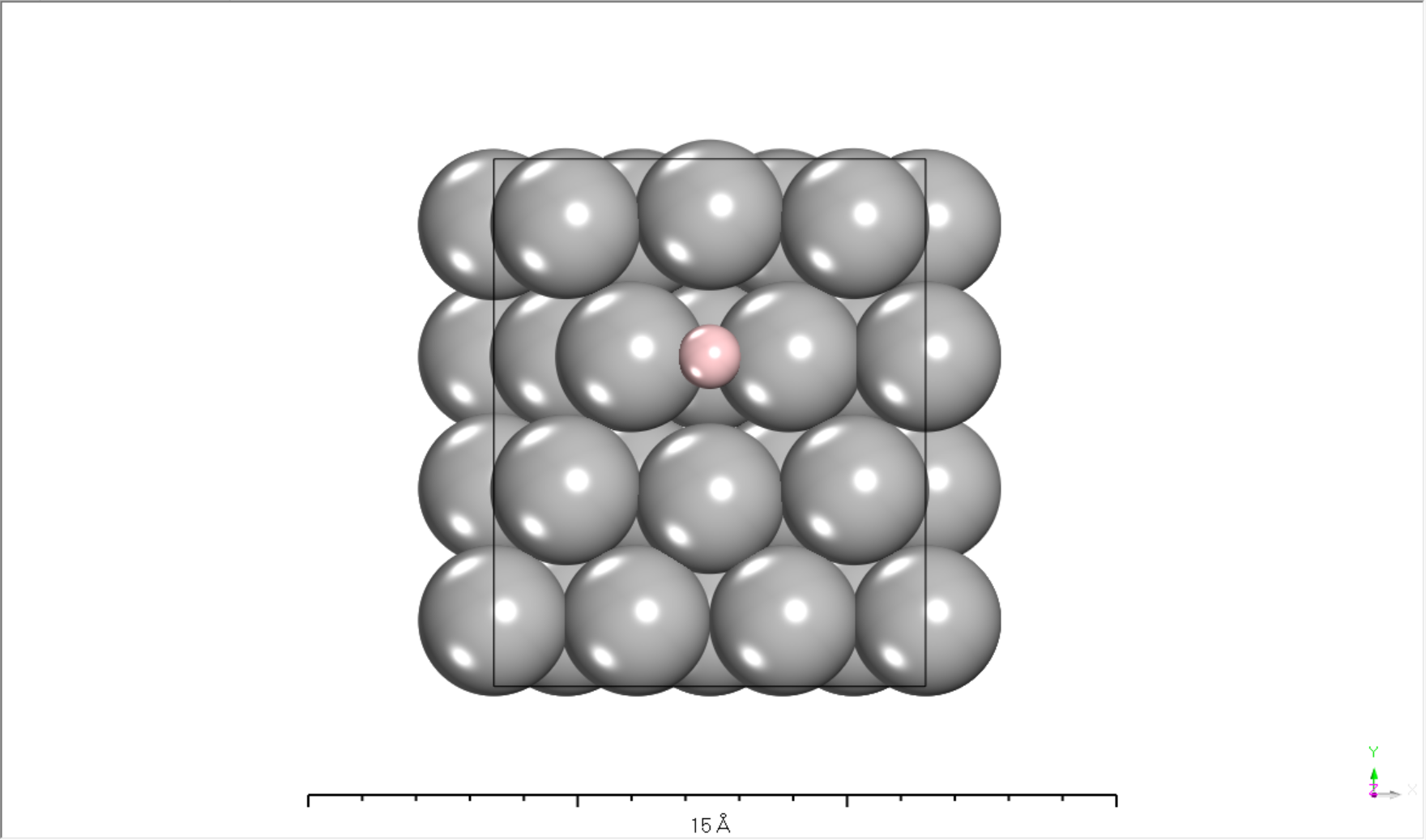 | Bridge | 0.392 |
| 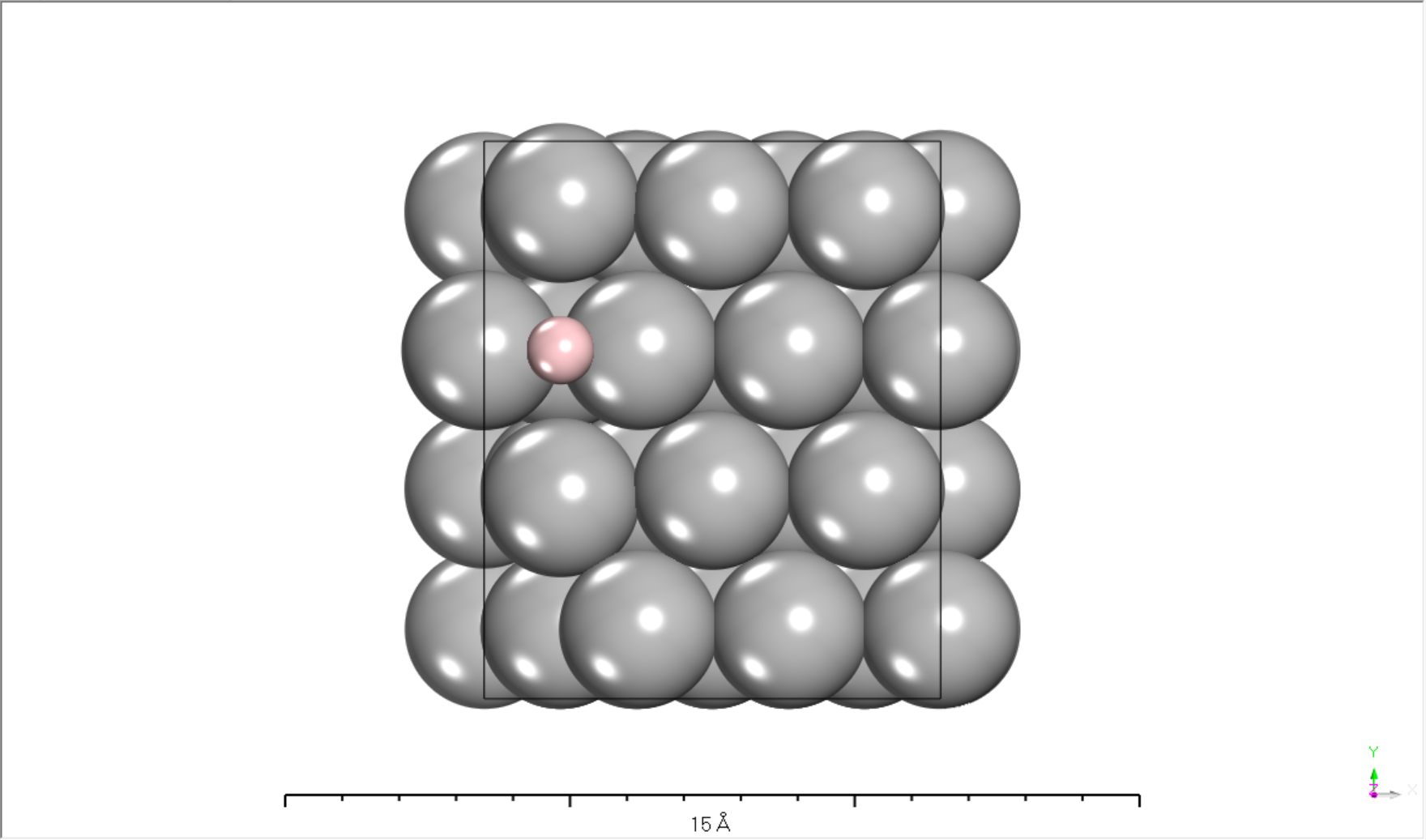 | Bridge | 0.458 |
| 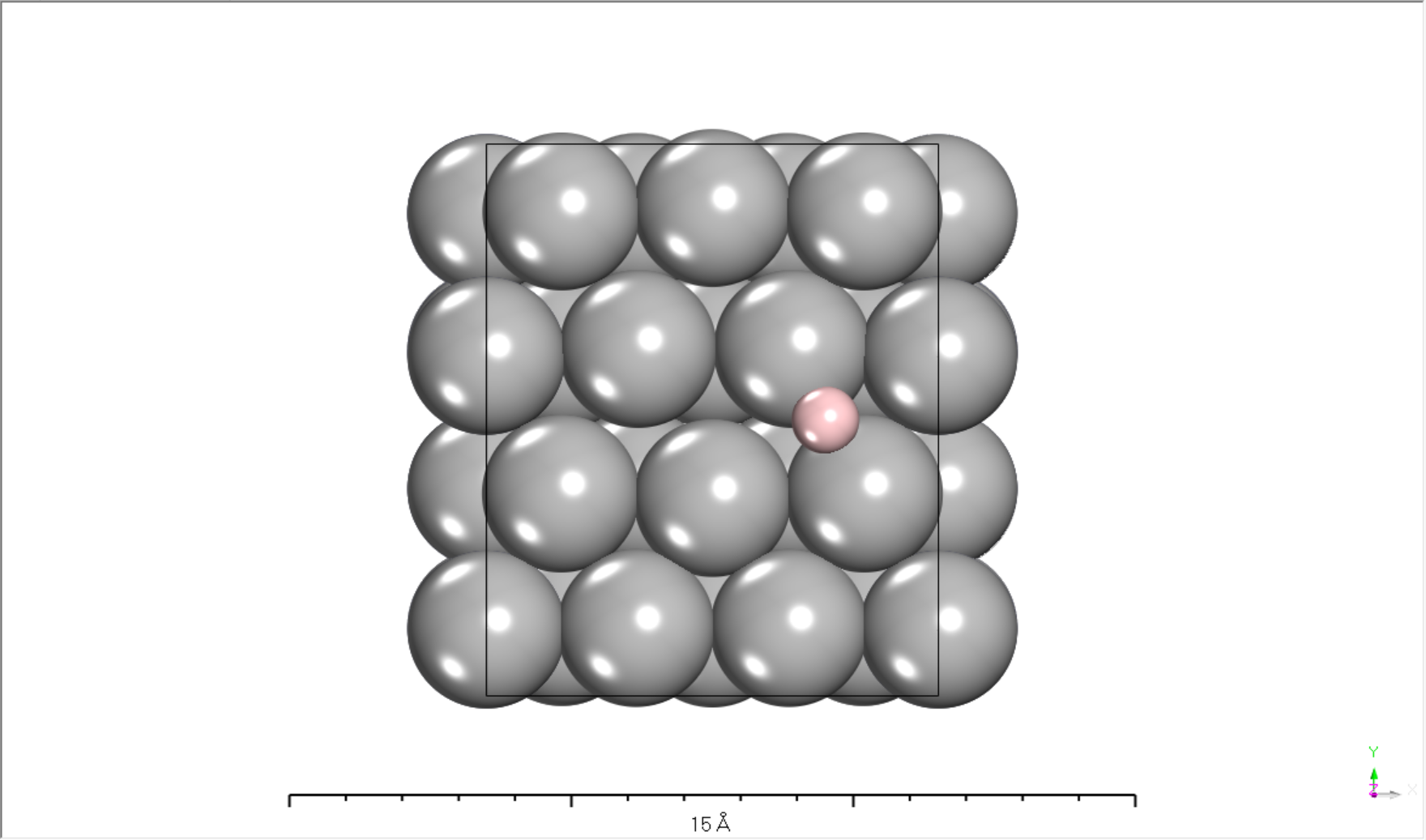 | Bridge | 0.357 |
| 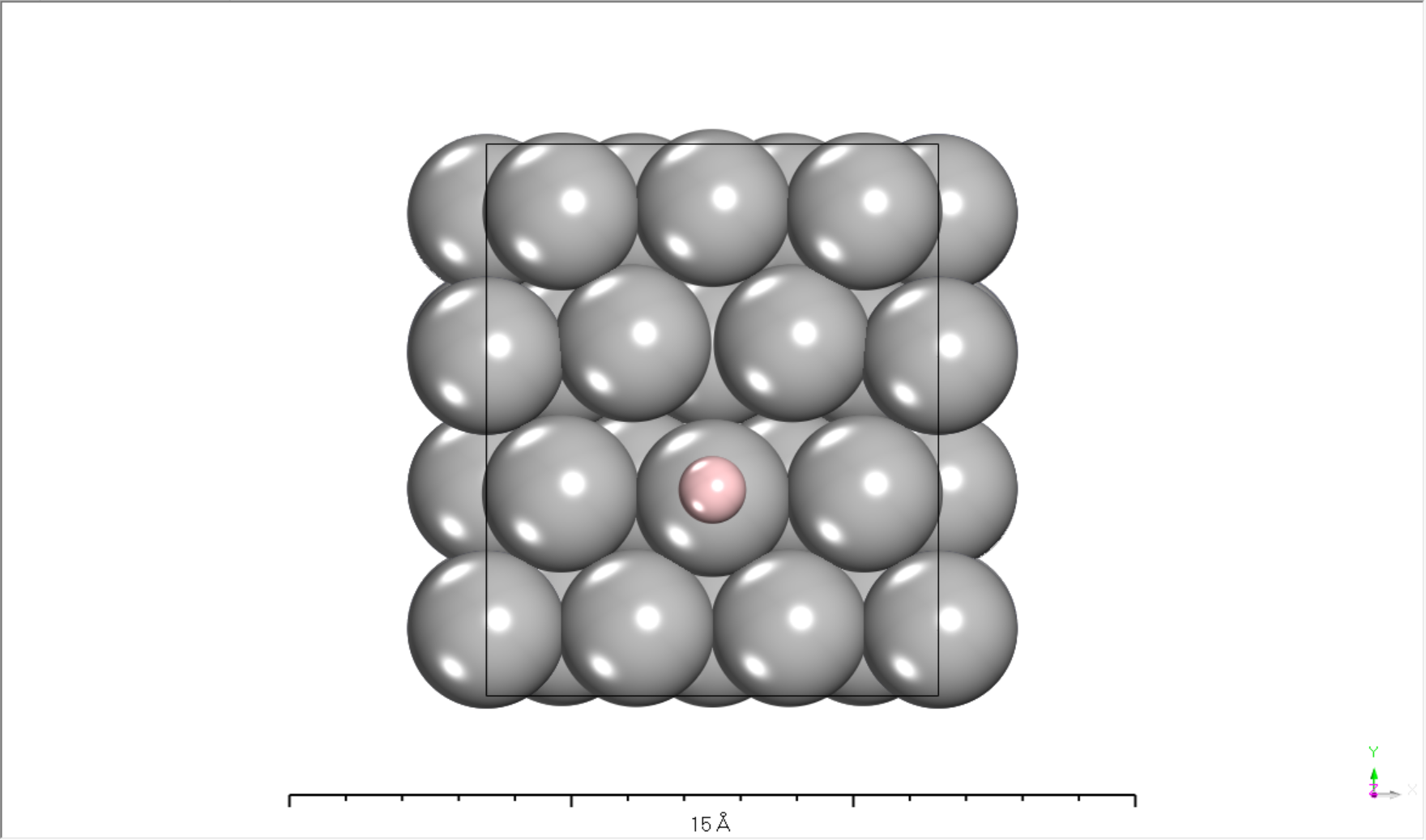 | Top | 0.652 |

**Table S7.** Details of Gibbs free energy correction for H adsorbed on (002) and (100) surface with different H coverages. n represent the number of adsorbed H atoms. ${ZPE}_{H^{*}}$ and ${TS}_{H^{*}}$ for n > 1 are the average ${ZPE}_{H^{*}}$ and ${TS}_{H^{*}}$ of n H* respectively.

| Miller indices | Adsorption site | *n* | H coverage ($Å^{-2}$) | $\Delta E_{H^{*}}$  (eV) | ${ZPE}_{H^{*}}$  (eV) | $TS_{H^{*}}$  (eV) | ${\frac{1}{2}\mathrm{ZPE}}_{H_{2}}$  (eV) | ${\frac{1}{2}TS}_{H_{2}}$  (eV) | $\Delta G_{H^{*}}$  $(eV)$ |
| --- | --- | --- | --- | --- | --- | --- | --- | --- | --- |
| (0 0 2) | top | 1 | 0.010 | 0.563 | 0.105 | 0.000 | 0.140 | 0.049 | 0.577 |
|  |  | 2 | 0.020 | 0.617 | 0.073 | 0.018 |  |  | 0.581 |
|  |  | 4 | 0.041 | 0.742 | 0.133 | 0.052 |  |  | 0.731 |
|  | bridge | 1 | 0.010 | 0.600 | 0.093 | 0.013 | 0.140 | 0.049 | 0.589 |
|  |  | 2 | 0.020 | 0.619 | 0.071 | 0.008 |  |  | 0.590 |
|  |  | 4 | 0.041 | 0.645 | 0.045 | 0.025 |  |  | 0.574 |
| (1 0 0) | bridge | 1 | 0.009 | 0.379 | 0.082 | 0.012 | 0.140 | 0.049 | 0.357 |
|  |  | 2 | 0.018 | 0.442 | 0.065 | 0.064 |  |  | 0.351 |
|  |  | 4 | 0.037 | 0.464 | 0.150 | 0.068 |  |  | 0.505 |
|  | hollow | 1 | 0.009 | 0.339 | 0.066 | 0.015 | 0.140 | 0.049 | 0.298 |
|  |  | 2 | 0.018 | 0.430 | 0.137 | 0.009 |  |  | 0.467 |
|  |  | 4 | 0.037 | 0.503 | 0.134 | 0.009 |  |  | 0.537 |

**Table S8.** Details of Gibbs free energy correction for H adsorbed on (101), (102), and (103) surface with different H coverages. n represent the number of adsorbed H atoms. ${ZPE}_{H^{*}}$ and ${TS}_{H^{*}}$ for n > 1 are the average ${ZPE}_{H^{*}}$ and ${TS}_{H^{*}}$ of n H* respectively.

| Miller indices | Adsorption site | *n* | H coverage ($Å^{-2}$) | $\Delta E_{H^{*}}$  (eV) | ${ZPE}_{H^{*}}$  (eV) | $TS_{H^{*}}$  (eV) | ${\frac{1}{2}\mathrm{ZPE}}_{H_{2}}$  (eV) | ${\frac{1}{2}TS}_{H_{2}}$  (eV) | $\Delta G_{H^{*}}$  $(eV)$ |
| --- | --- | --- | --- | --- | --- | --- | --- | --- | --- |
| (1 0 1) | bridge | 1 | 0.008 | -0.041 | 0.164 | 0.010 | 0.140 | 0.049 | 0.023 |
|  |  | 2 | 0.017 | -0.034 | 0.165 | 0.014 |  |  | 0.026 |
|  |  | 4 | 0.033 | 0.004 | 0.167 | 0.015 |  |  | 0.065 |
| (1 0 2) | bridge | 1 | 0.009 | 0.107 | 0.168 | 0.011 | 0.140 | 0.049 | 0.174 |
|  |  | 2 | 0.018 | 0.109 | 0.173 | 0.010 |  |  | 0.181 |
|  |  | 6 | 0.055 | 0.142 | 0.261 | 0.013 |  |  | 0.217 |
| (1 0 3) | bridge | 1 | 0.007 | -0.101 | 0.152 | 0.013 | 0.140 | 0.049 | -0.054 |
|  |  | 2 | 0.015 | -0.003 | 0.151 | 0.014 |  |  | 0.043 |
|  |  | 4 | 0.030 | 0.047 | 0.155 | 0.015 |  |  | 0.096 |

**Table S9.** The Bader charge of surface Zn near the adsorption sites with the lowest Gibbs free energy.

| Miller indices | Bader charge of surface Zn (e) | Net charge of surface Zn (e) |
| --- | --- | --- |
| (0 0 2) | 11.972 | -0.028 |
| (1 0 0) | 11.977 | -0.023 |
| (1 0 1) | 12.000 | 0.000 |
| (1 0 2) | 11.993 | -0.007 |
| (1 0 3) | 12.002 | 0.002 |
